# LIPUS Promotes Calcium Oscillation and Enhances Calcium Dependent Autophagy of Chondrocytes to Alleviate Osteoarthritis

**DOI:** 10.1002/advs.202413930

**Published:** 2025-02-27

**Authors:** Mengtong Guan, Xiaoyu Han, Bo Liao, Wang Han, Lin Chen, Bin Zhang, Xiuqin Peng, Yu Tian, Gongyi Xiao, Xinhe Li, Liang Kuang, Ying Zhu, Dingqun Bai

**Affiliations:** ^1^ Department of Rehabilitation Medicine Key Laboratory of Physical Medicine and Precision Rehabilitation of Chongqing Municipal Health Commission The First Affiliated Hospital of Chongqing Medical University Chongqing 400010 China; ^2^ State Key Laboratory of Ultrasound in Medicine and Engineering Chongqing Medical University Chongqing 400016 China; ^3^ Center of Bone Metabolism and repair laboratory for Prevention and rehabilitation of Training injuries State Key laboratory of Trauma Burns and combined injury Trauma center Research Institute of Surgery Daping Hospital Army Medical University (Third Military Medical University) Chongqing 400000 China; ^4^ Department of Orthopedics Chonggang General Hospital Chongqing 400000 China

**Keywords:** autophagy, calcium signaling, chondrocytes, low‐intensity pulsed ultrasound, osteoarthritis

## Abstract

Osteoarthritis (OA) is a degenerative disease which places an enormous burden on society, effective treatments are still limited. As a non‐invasive and safe physical therapy, low‐intensity pulsed ultrasound (LIPUS) can alleviate OA progression, but the underlying mechanism is not fully understood, especially the mechanical transduction between LIPUS and the organism. In this pioneering study, the biomechanical effects of LIPUS on living mice chondrocytes and living body zebrafish are investigate by using fluorescence imaging technology, to dynamically “visualize” its invisible mechanical stimuli in the form of calcium oscillations. It is also confirmed that LIPUS maintains cartilage homeostasis by promoting chondrocyte autophagy in a calcium‐dependent manner. In addition, chondrocyte ion channels are screened by scRNA‐seq and confirm that the mechanosensitive ion channel transient receptor potential vanilloid 4 (TRPV4) mediated the biological effects of LIPUS on chondrocytes. Finally, it is found that a combination of pharmacologically induced and LIPUS‐induced Ca^2+^ influx in chondrocytes enhances the cartilage‐protective effect of LIPUS, which may provide new insights for optimizing LIPUS in the treatment of OA.

## Graphical abstract

A non‐invasive and safe physical therapy, low‐intensity pulsed ultrasound (LIPUS) can alleviate osteoarthritis. This study dynamically “visualizes” the invisible mechanical stimuli of LIPUS on living chondrocytes by fluorescence imaging technology in the form of calcium oscillations, confirming LIPUS maintains cartilage homeostasis by promoting chondrocyte autophagy in a calcium‐dependent manner. Mechanosensitive ion channel TRPV4 mediates this effect.

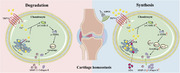

## 1 Introduction

Osteoarthritis (OA) is a degenerative disease involving the whole joint. Destruction of cartilage is the key pathological change of OA, which was manifested by cartilage thinning, loss of proteoglycans, vertical clefts/erosion to the calcified cartilage and abnormal cartilage calcification.^[^ ^1^, ^2^ ^]^ In addition, it is usually accompanied by synovitis, subchondral bone remodeling, and osteophyte formation.^[^ ^3^ ^]^ The classical symptoms and signs of OA include pain, stiffness, malfunction of joint motion, which affected 7% of the global population, more than 500 million people worldwide.^[^ ^4^, ^5^, ^6^ ^]^ However, due to the complicate pathogenesis of OA, current therapies mainly focus on pain control in early OA or total knee joint replacement for late osteoarthritis.^[^ ^7^ ^]^ There is a lack of effective treatment for alleviating OA progression.

Low‐intensity pulsed ultrasound (LIPUS) is a kind of pulsed acoustic pressure wave that can provide localized mechanical stimulus to cells, which affects cell biology functions via regulating several molecular and cellular pathways.^[^ ^8^, ^9^ ^]^ As an effective and non‐invasive rehabilitation treatment, LIPUS is of anti‐inflammatory and pro‐repair effects which could be a cost‐effective alternate for early OA prevention. Previous clinical studies showed that LIPUS is effective in OA pain relief and joint functional improvement.^[^ ^9^, ^10^, ^11^ ^]^ The underling mechanism could be attributed to LIPUS maintained cartilage matrix homeostasis through NF‐Κb,^[^ ^8^ ^]^ FAK,^[^ ^12^ ^]^ ZNT‐9,^[^ ^13^ ^]^ and VEGFA^[^ ^14^ ^]^ signaling pathways. However, the mechanisms through which LIPUS mechanical stimuli translating into a chondrocyte's biological effects are not well understood, which hinders the elucidation of the dose‐effect relationship of LIPUS and the exploration of how to improve the therapeutic efficacy of LIPUS.

Chondrocyte is the only cell type in normal cartilage, enclosed in a dense extracellular matrix (ECM), which plays vital roles in maintaining the biological and mechanical functions of cartilage.^[^ ^1^ ^]^ Chondrocytes are mechanosensitive cells, that integrate mechanical stimuli into cellular responses in a process known as mechanotransduction.^[^ ^15^ ^]^ They sense changes in mechanical loading and respond by adjusting either anabolic or catabolic processes to maintain the structure and compliance of the tissue.^[^ ^16^ ^]^ Which due to the abundant expression of mechanically‐activated (MA) ion channels, such as Piezo1, Piezo2, and transient receptor potential (TRP) family channels.^[^ ^17^, ^18^, ^19^, ^20^ ^]^ Mechanical stimuli activate MA channels for a rapid influx of Ca^2+^, which occurs in seconds to minutes,^[^ ^20^, ^21^ ^]^ together with calcium‐induced calcium release from intracellular calcium stores (usually the endoplasmic reticulum), causing a sudden increasing and subsequent recovery of cytosolic Ca^2+^ concentration. The rapid change of cytosolic Ca^2+^ concentration is defined as calcium oscillation.^[^ ^22^, ^23^ ^]^ The cytosolic Ca^2+^ functions as a second messenger to influence biology effect of chondrocytes, such as cell metabolism,^[^ ^19^, ^24^ ^]^ adhesion behavior^[^ ^25^ ^]^ and autophagy.^[^ ^26^, ^27^, ^28^ ^]^ Particularly, the Ca^2+^ channel transient receptor potential vanilloid 4 (TRPV4) has been shown to participate in OA progression by regulating chondrocyte differentiation and matrix homeostasis, including promoting SOX9, collagen II, and aggrecan expression in chondrocytes.^[^ ^29^, ^30^ ^]^ Sensation of mechanical stimuli by chondrocytes is important for cartilage homeostasis.^[^ ^17^ ^]^ Optimal mechanical loading enhances chondrogenesis^[^ ^31^ ^]^ and is effective in prevention OA.^[^ ^32^ ^]^ Combined with these studies, we hypothesis that mechanical stimulation of LIPUS is a beneficial stimulus for chondrocytes and is somewhat related to calcium signaling.

Autophagy is a highly conserved degradation process in all eukaryotic cells,^[^ ^33^ ^]^ which has been shown to maintain the function of chondrocytes and alleviate OA progression.^[^ ^34^ ^]^ Impairment of autophagy by specific knockout of Atg5 (a key autophagy‐related gene) in chondrocytes promoted age‐related OA in mice.^[^ ^35^ ^]^ When autophagic flux inhibition, the osteoarthritic chondrocytes secreted the calcified extracellular vesicles (EVs) to the ECM, the microtubule‐associated proteins light chain 3 (LC3)‐conjugated calcified EVs form the mineral nodule to aggravate calcification of the osteoarthritic cartilage.^[^ ^36^ ^]^ On the other hand, enhancing cartilage autophagy by specific deletion of mTOR (a major repressor of autophagy)^[^ ^37^ ^]^ or using exosomes induced‐mTOR inhibition protected mice from destabilization of the medial meniscus (DMM)‐induced osteoarthritis.^[^ ^38^ ^]^ Therefore, chondrocyte autophagy plays a critical role in OA progression and modulating autophagy could be a promising strategy for OA treatment.

In this study, we established specific experimental systems which were used to dynamically image the calcium oscillations (and autophagic flux) of chondrocytes under (and after) the treatment of LIPUS. We revealed that LIPUS could instantly activate calcium signaling of chondrocytes in vitro and in living zebrafish which induced significant autophagic changes after LIPUS treatment. This is a pioneer visualization of LIPUS‐induced mechanical and biological effect on chondrocyte. We confirmed that LIPUS could promote chondrocyte autophagy in a calcium‐dependent manner. Furthermore, we clarified that TRPV4, a mechanosensitive ion channel which is highly expressed in chondrocyte, mediated the calcium activation and upregulation of autophagy by LIPUS. Finally, we found that pharmacological induction and LIPUS‐induced chondrocyte calcium oscillations had a synergistic effect and further enhanced the chondroprotective effect of LIPUS, which may bring new insight for optimizing LIPUS in OA prevention.

## 2 Results

### 2.1 LIPUS Activates Ca^2+^ Signaling of Chondrocytes

**Figure 1:**
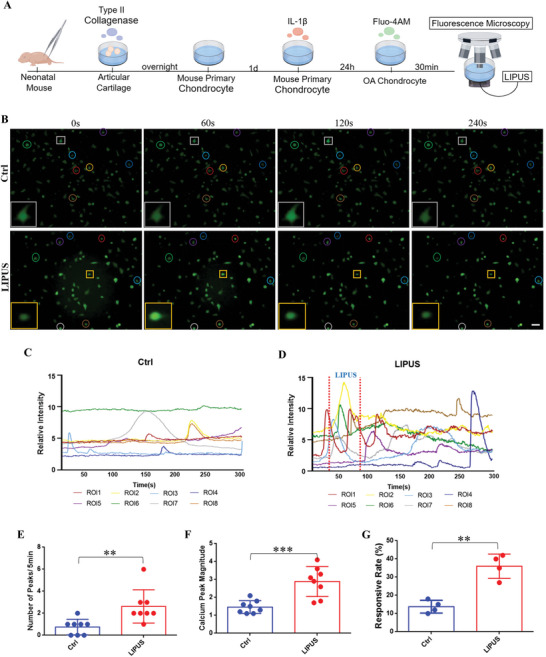
LIPUS activates Ca^2+^ signaling of inflammatory chondrocyte. A) Schematic of the inflammatory chondrocyte establishment and the real‐time calcium imaging with LIPUS. B) The real‐time fluorescence intensity images of chondrocyte at different time point in the same field of view (Circles of the same color represent the same cell, and square represent the typical cell in this field of view). Scale bar, 10 µm. Calcium transient relative fluorescence intensity of chondrocyte without C) and with D) LIPUS. Quantification of the number E) (*n* = 8), the magnitude F) (*n* = 8) of calcium peaks, and the responsive rate of cells G) (*n* = 4). Data are presented as means ± SD. Statistical analysis was performed using Student's t test. ***(*P* < 0.001), **(*P* < 0.01).

As a type of intrinsically mechanosensitive cell, the intracellular calcium ([Ca^2+^]i) is among the earliest and most fundamental signals in response to physical stimuli.^[^ ^21^, ^39^ ^]^ To visualize the invisible LIPUS stimulation as dynamic biological effect, we employed a customized experimental setup to observe the real‐time calcium signaling of chondrocytes under the treatment of LIPUS (**Figure** **1**A; Figure S1A, Supporting Information). We established the inflammatory chondrocyte model by stimulating cultured mouse primary chondrocytes with interleukin‐1beta (IL‐1β), as reported previously.^[^ ^8^, ^14^, ^40^ ^]^ The real‐time images of cells were shown in (Figure 1B; and Movies S1 and S2, Supporting Information). In the absence of LIPUS treatment, calcium probe fluorescence signals showed that inflammatory chondrocytes were accompanied by only episodic and low‐spoke calcium oscillations, whereas in the LIPUS‐treated group, with classical treatment parameters (ultrasound frequency 1.5 MHz; pulse repetition frequency: 1.0 KHz; intensity 30 mW cm^−2^; duty cycle 20%),^[^ ^8^, ^14^, ^41^ ^]^ exhibited significantly enhanced calcium oscillations. Each curve, corresponding to a specific color, represented the relative fluorescence intensity of calcium transients in individual cells within the control and LIPUS groups (Figure 1C,D). In the control group, we observed that inflammatory chondrocytes exhibited asynchronous spontaneous calcium oscillations, while the responding curve were relatively flat. In the LIPUS group, the fluorescence intensity traces displayed several prominent peaks compared with the baseline during stimulating (between the two red lines). After LIPUS treatment, some calcium peaks gradually returned to baseline, while a few cells exhibited delayed peaks. Statistical analysis revealed that LIPUS nearly tripled the frequency of calcium oscillations (Figure 1E) and doubled the magnitude (Figure 1F). Additionally, the proportion of calcium‐responsive chondrocytes significantly increased in response to LIPUS (Figure 1G). These results demonstrate that LIPUS induces calcium oscillations in inflammatory chondrocytes in vitro.

**Figure 2:**
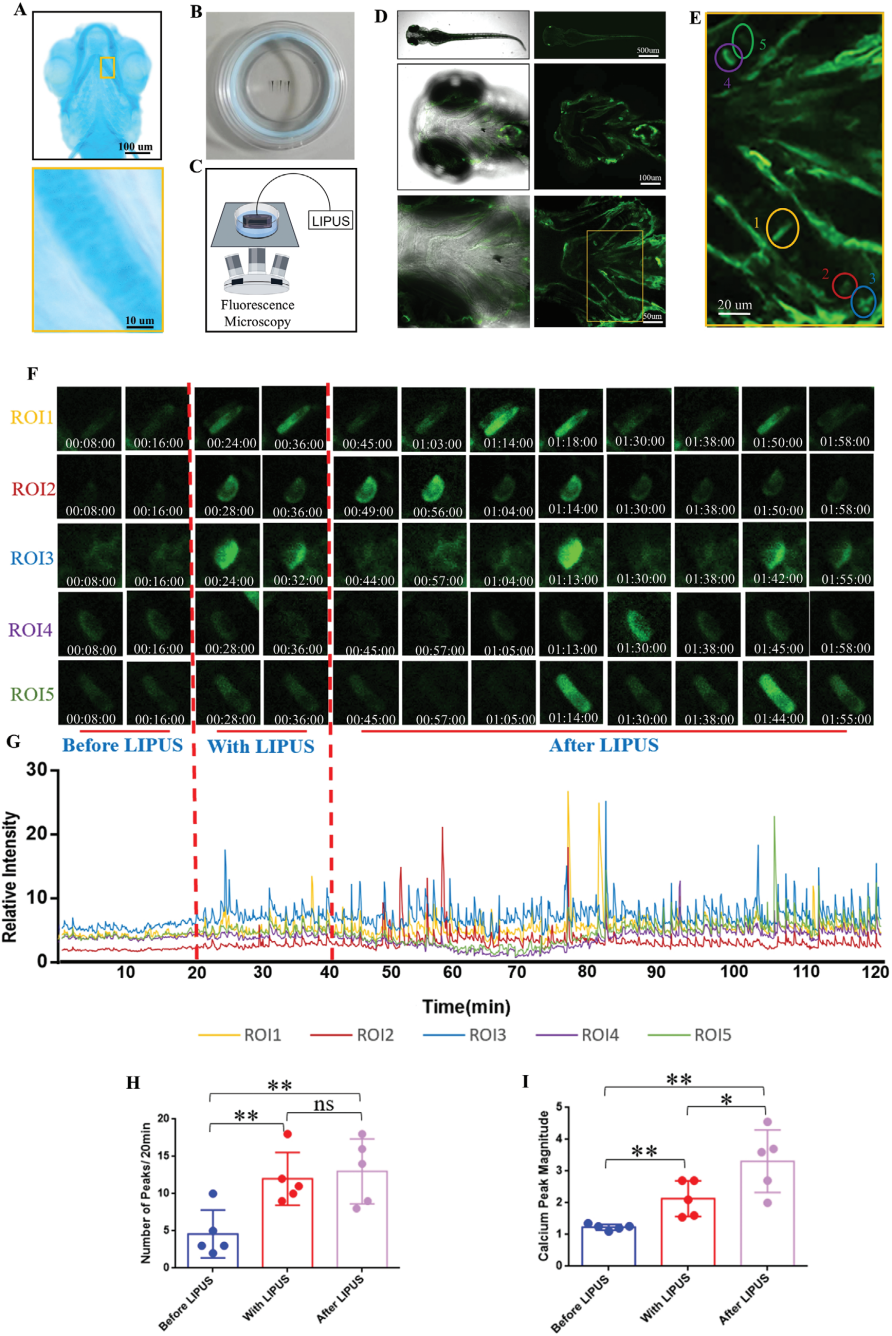
LIPUS promotes chondrocyte calcium oscillation of living zebrafish. A) Alcian blue staining to label the cranial cartilage structure of zebrafish juveniles. B) Zebrafish larvae were immobilized in a confocal dish with low‐melting‐point agarose. C) Schematic of the real‐time LIPUS‐processed calcium imaging under a confocal microscope. D) The whole fish, cranial and ceratohyal observed under a confocal microscope. E) 5 chondrocytes were circled with different colors as ROIs. F) The real‐time fluorescence intensity of the five ROIs. G) Calcium transient relative fluorescence intensity of ROIs. Quantification of the number H) (*n* = 5), the magnitude I) (*n* = 5) of calcium peaks. Data are presented as means ± SD. Statistical analysis was performed using Student's t test. **(*P* < 0.01), *(*P* < 0.05), ns (0.05 < P).

To further investigate the biological effects of LIPUS in vivo, we used a zebrafish larval model to demonstrate the activation of calcium signaling in chondrocyte. Zebrafish, a model animal with high homology to the human genome, is advantageous for confocal microscopy due to the small, translucent cranium of juveniles, which is less than 1 mm thick. The earliest detectable bony structure, the cranial cartilage, appears at 3 days post fertilization (dpf). It is well developed between 5 and 10 dpf, with the complete structure observable.^[^ ^42^ ^]^ We selected juveniles at 5 dpf for subsequent observation. Using the common method of alcian blue staining for cartilage morphological analysis,^[^ ^42^, ^43^ ^]^ we labeled the cranial cartilage structure of zebrafish juveniles. The contours of chondrocytes can be viewed by magnifying the ceratohyal cartilage (**Figure** **2**A) and this area was observed under confocal microscopy subsequently. The GCaMP6s transgenic zebrafish line, known for its high sensitivity and stability, enables dynamic monitoring of intracellular calcium concentration.^[^ ^44^ ^]^ We further used 5dpf GCaMP6s zebrafish juveniles to perform real‐time LIPUS‐processed calcium imaging under a confocal microscope (Figure 2B,C, Figure S1B,C, Supporting Information). The whole fish, cranial and ceratohyal were shown in (Figure 2D), 5 clearly visible chondrocytes were circled with different colors as regions of interest (ROIs) for subsequently quantifying real‐time fluorescence intensity (Figure 2E). The entire imaging results were displayed in (Movie S3, Supporting Information). The real‐time fluorescence intensity of the five ROIs were shown in (Figure 2F), and these data were quantified to create the calcium oscillation curve shown in (Figure 2G). The results showed that there were only occasional and low magnitude calcium peaks in ROIs before LIPUS treatment. During LIPUS stimulation, the calcium peaks gradually became apparent, and the peak amplitude increased. Interestingly, the calcium oscillation in ROIs did not stop with the cessation of LIPUS treatment, moreover the amplitude of the calcium peak increased to a certain extent (Figure 2H,I). This suggests LIPUS activates calcium signaling of chondrocytes in living organism, inducing a sustained effect. In summary, we have turned LIPUS‐mediated invisible mechanical effects into visible and quantifiable calcium oscillatory signals in living chondrocytes for the first time, revealing the mechanism of action of chondrocytes in response to LIPUS treatment, laying an important foundation for understanding LIPUS‐mediated mechanical signaling.

### 2.2 LIPUS Up‐Regulates Chondrocyte Autophagy in a Calcium‐Dependent Manner

**Figure 3:**
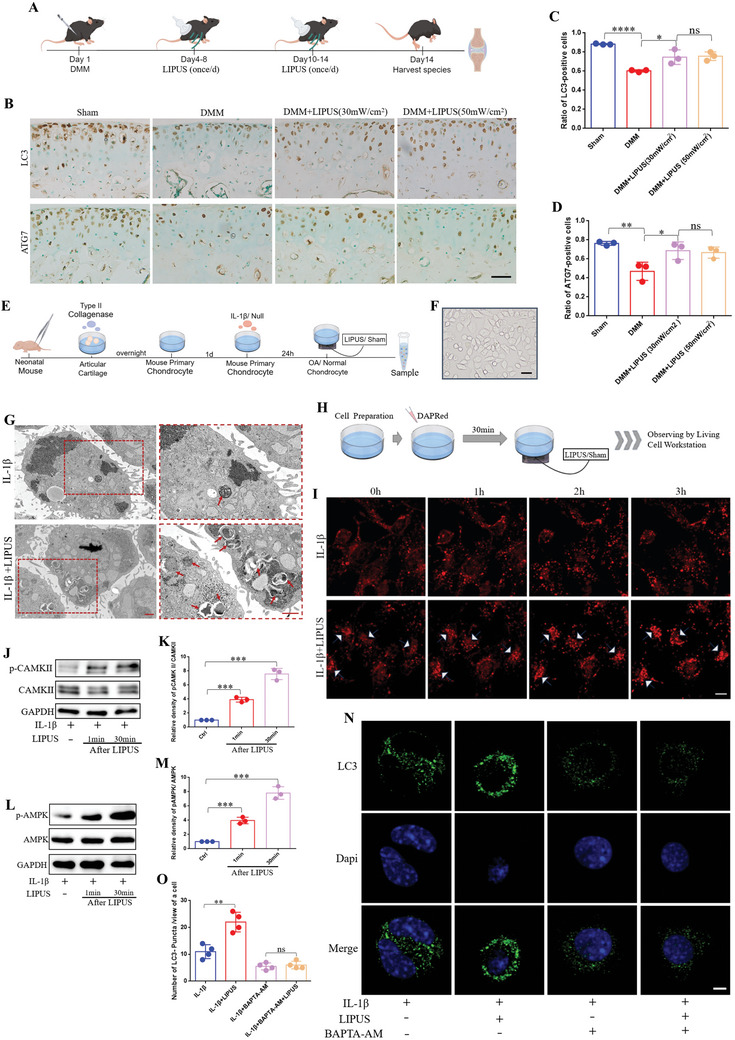
LIPUS up‐regulates chondrocyte autophagy in a calcium pathway‐dependent manner. A) Schematic of the mouse experiment procedures. B) Immunohistochemical staining of LC3 and ATG7 in the articular cartilage. Scale bar, 50 µm. Quantification of LC3‐positive cells C) and ATG7‐positive cells D) (*n* = 3 mice). E) Schematic of the inflammatory chondrocyte establishment and treating with LIPUS. F) The chondrocytes we have obtained observing by microscope. Scale bar, 20µm. G) TEM was used for detection the autophagosomes in chondrocytes following LIPUS treatment. Red arrowhead indicates an autophagosome. Scale bar, 1 µm. H) Observation the autophagy flux of living chondrocyte labeled by DAPRed. I) Autophagy flux was dynamically observed by DAPRed at different time point after LIPUS. White arrowhead indicates the significant changes. Scale bar, 10 µm. WB (J) and densitometry analysis (K) of CAMK II protein expression following LIPUS treatment (*n* = 3). WB L) and densitometry analysis M) of AMPK protein expression following LIPUS treatment at different time point (*n* = 3). N) Immunofluorescence was used to detect the LC3‐puncta after the chondrocytes was co‐incubated with BAPTA‐AM. Scale bar, 5 µm. O) Quantification of LC3‐puncta in chondrocytes (*n* = 4). Data are presented as means ± SD. Statistical analysis was performed using Student's t test. ****(*P* < 0.0001), ***(*P* < 0.001), **(*P* < 0.01), *(*P* < 0.05), ns (0.05 < P).

Autophagy is a crucial mechanism for sustaining cartilage homeostasis.^[^ ^34^, ^35^, ^36^, ^37^, ^38^ ^]^ Previous studies have revealed LIPUS up‐regulates autophagy of macrophages,^[^ ^45^ ^]^ mesenchymal stem cells (MSCs)^[^ ^46^ ^]^ in attenuating osteoarthritis. Thus, we investigated the effect of LIPUS on chondrocyte autophagy. First, we conducted DMM surgery on the right knee joint of 9‐week‐old male C57BL/6J mice, a well‐established model for traumatic OA.^[^ ^47^ ^]^ Post‐surgery, the mice were subjected to a 2‐week LIPUS treatment before sacrificed (**Figure** **3**A). Since DMM models predominantly feature synovial inflammation within the first two weeks. As shown in the HE staining, the synovial inflammation of injured joints was more severe than that of control mice, after LIPUS treatment, the synovitis was alleviated (Figure S2A, Supporting Information). We also assessed the total synovitis scores according to synovitis scoring system described before.^[^ ^48^ ^]^ The scores in DMM surgery mice were remarkably higher compared that of the negative control mice and the scores were lower in the LIPUS group (Figure S2B, Supporting Information). Moreover, LIPUS treatment significantly decreased the expressing of iNOS in the synovium of mice with DMM surgery (Figure S2C,D, Supporting Information). Collectively, these results indicate that LIPUS alleviates the synovitis of mice with DMM surgery, which corresponds with our previous study^[^ ^45^ ^]^ and indicates that the model was successfully established. In addition, DMM surgery did not cause a change of cartilage thickness at this time point (Figure S2E–G, Supporting Information). What we would like to highlight is immunohistochemical analysis revealed decreased expression of autophagy markers LC3 and ATG7 in the cartilage of the DMM group relative to the sham group. Furthermore, LIPUS with the intensity both of 30 and 50 mW cm^−^^2^ elevated the protein level of LC3 and ATG7, although no significant difference was observed between the two intensities (Figure 3B–D). This demonstrated LIPUS restores the impaired autophagy capacity of chondrocyte in DMM‐induced OA mice, highlighting its potential therapeutic role in osteoarthritis management.

To clarify whether LIPUS directly regulates chondrocyte autophagy, we replicated the in vitro inflammatory chondrocyte model and subsequently treated it with LIPUS (Figure 3E). Given that autophagy is a dynamic process, we collected cellular proteins at various time points post‐LIPUS treatment. Western blot (WB) analysis revealed a significant accumulation of LC3‐II (the critical components of autophagosomes) at 2 h post‐LIPUS treatment (Figure S3A,B, Supporting Information), which was adopted as the observation time point for subsequent experiments. We also examined the effects of varying LIPUS intensities (30, 40, and 50 mW cm^−2^) on chondrocyte autophagy. The results indicated that all intensities upregulated autophagy levels relative to the IL‐1β group, yet no significant differences were observed (Figure S3C,D, Supporting Information), thus, we chose the 30 mW cm^−^^2^ intensity as previously. Following the determination of the optimal time point and LIPUS intensity, transmission electron microscopy (TEM) data showed an increase of autophagosomes in LIPUS‐treated chondrocytes (Figure 3G). In addition, the red fluorescent LC3‐puncta were also dramatically increased in chondrocytes treated by LIPUS (Figure S3E,F, Supporting Information). These findings suggest that LIPUS directly enhanced chondrocyte autophagy.

As the accumulation of LC3‐II can result from either enhanced autophagic flux or decreased autophagic degradation.^[^ ^49^ ^]^ In order to dynamically observe the effect of LIPUS on autophagy flux. We performed a 3 h live‐cell imaging study to monitor autophagy flux in chondrocytes, employing the red‐fluorescent autophagic probe DAPRed (Dojindo, Japan) (Figure 3H), which recognizes various autophagic vacuoles and shows high photostability in living cells.^[^ ^50^ ^]^ The results provided clear evidence that LIPUS enhances autophagy flux in chondrocytes. Initially, the red fluorescence in the IL‐1β group was diffused without significant change; however, after LIPUS stimulation, the fluorescence gradually intensified, enlarged, and coalesced into clusters (Figure 3I; and Movies S4 and S5, Supporting Information). Additionally, we transfected primary chondrocytes with a dual‐tagged LC3 [red fluorescence protein (RFP)‐green fluorescence protein (GFP)‐LC3] adenovirus to assess the impact of LIPUS on autophagic flux.^[^ ^51^ ^]^ The number of autolysosomes labeled by RFP and the autophagosomes labeled by GFP in IL‐1β‐treated chondrocytes increased with LIPUS treatment (Figure S3G, Supporting Information). Ultimately, blocking autolysosome degradation with bafilomycin A1 (BafA1), LIPUS further promoted the accumulation of LC3‐II in BafA1‐pretreated chondrocytes (Figure S3H,I, Supporting Information). These findings are solid evidence of LIPUS directly enhances autophagy flux of chondrocytes in vitro.

To further elucidate the relationship between LIPUS‐induced calcium oscillations and the upregulation of chondrocyte autophagy, we examined the Ca^2+^‐associated signaling molecule calcium–calmodulin dependent protein kinase (CaMK II), along with its downstream effector AMP‐activated protein kinase (AMPK), both of which are implicated in the regulation of autophagy. As shown in the WB, LIPUS significantly increased the phosphorylation levels of CAMK II and AMPK, with the most pronounced effects observed 30 min post‐treatment cessation (Figure 3J–M). Conversely, pretreatment of chondrocytes with the intracellular calcium chelator BAPTA‐AM^[^ ^52^ ^]^ substantially diminished the autophagy‐enhancing effect of LIPUS (Figure 3N,O). This result suggested that LIPUS could regulate chondrocyte autophagy through Ca^2+^‐mediated CAMK II‐AMPK signaling pathway. In summary, LIPUS up‐regulates chondrocyte autophagy in a calcium‐dependent manner.

### 2.3 LIPUS Maintains Cartilage Homeostasis Depending on Autophagy

**Figure 4:**
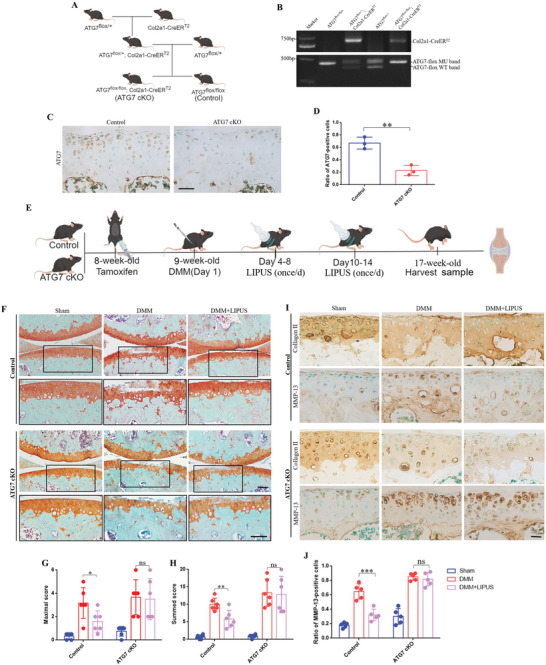
LIPUS maintains cartilage homeostasis depending on autophagy. A) Reproductive strategies for transgenic mice. B) Genotyping by PCR to confirm the Cre recombinase and homozygous floxed ATG7 in product. C)ATG7 deletion was confirmed by immunohistochemistry of the articular cartilage. Scale bar, 50 µm. D) Quantification of ATG7‐positive cells in the articular cartilage (*n* = 3 mice). E) Schematic of the transgenic mice experiment procedures. F) Safranin O–Fast Green staining of the knee joint after LIPUS in ATG7cKO and control mice. Scale bar, 100 µm. The joint degradation was evaluated histologically by the OARSI recommended scoring system. Maximal scores G) and summed score H) were calculated for the MTP (*n* = 6 mice). I) Immunohistochemical staining of Collagen II and MMP‐13 in the articular cartilage of ATG7cKO and control mice. Scale bar, 20 µm. J) Quantification of MMP‐13‐positive cells (*n* = 5 mice). Data are presented as means ± SD. Statistical analysis was performed using Student's t test. ***(*P* < 0.001), **(*P* < 0.01), *(*P* < 0.05), ns (0.05 < P).

It has been established that chondrocyte autophagy plays a critical role in OA progression.^[^ ^34^, ^35^, ^36^, ^37^, ^38^, ^52^, ^53^, ^54^ ^]^ Consequently, we hypothesized that the alleviation of OA progression by LIPUS might be associated with its regulatory effect on chondrocyte autophagy. We clarified the effect of LIPUS on OA progression at autophagy‐deficient statements both in vivo and in vitro. ATG7, integral to chondrocyte autophagy, is implicated in cartilage development and OA progression.^[^ ^55^, ^56^ ^]^ Initially, we generated the mice with a conditional deletion of ATG7 gene in chondrocytes, utilizing Col2a1‐CreERT2 mice, where Cre recombinase expression is under the control of the chondrocyte‐specific Col2a1 promoter, inducible by Tamoxifen (TM).^[^ ^57^ ^]^ As shown in (Figure **4**A), the ATG7 conditional knockout mice (ATG7^flox/flox^ Col2a1‐CreER^T2^, ATG7cKO) and control mice (ATG7^flox/flox^, Control) were generated, genotypes were ascertained by PCR analysis (Figure 4B). Immunohistochemistry post intraperitoneal injection of TM verified the efficient knockout of ATG7 in cartilage (Figure 4C,D), confirming successful knockdown. We examined the therapeutic role of LIPUS in OA progression following the steps outlined in (Figure 4E). Safranin O–Fast Green staining revealed a significant reduction in proteoglycan loss and articular cartilage degeneration in control mice treated with LIPUS compared with DMM group. Notably, the therapeutic effect was markedly diminished in ATG7cKO mice (Figure 4F). The summed and maximal Osteoarthritis Research Society International (OARSI) scores^[^ ^2^ ^]^ of the tibiae further demonstrated that LIPUS therapeutic effect was attenuated in ATG7cKO mice (Figure 4G,H). In addition, we assessed the expression of Collagen II and matrix metalloproteinase‐13 (MMP‐13), the key components and enzymes of cartilage matrix, respectively, which are indicative of cartilage matrix status.^[^ ^1^, ^58^ ^]^ The IHC results showed that the Collagen II protein level was significantly decreased whereas the positive cell rate of MMP13 was remarkably increased after DMM surgery both in Control and ATG7 cKO mice. In addition, the expression patterns of Collagen II and MMP‐13 post‐LIPUS treatment were reversed in control mice, but no significant alterations were observed in ATG7cKO mice (Figure 4I,J). These results suggested that the therapeutic effect of LIPUS is dependent on chondrocyte autophagy.

To further investigate the effect of LIPUS‐enhanced autophagy on maintaining the balance of extracellular matrix anabolic and catabolic. We blocked the autophagy flux by pretreating chondrocyte with Bafilomycin A1 (BafA1). WB as shown in (Figure S4A–C, Supporting Information), there was no significant difference of Collagen II or MMP‐13 level in BafA1 pretreated cells between IL‐1β and IL‐1β+LIPUS groups. Immunofluorescence shown a consistent result (Figure S4D–F, Supporting Information). So as in the toluidine blue staining, whose shade of color represents the synthesized of extracellular matrix. Chondrocytes treated with LIPUS were much darkly colored than those without LIPUS. While in BafA1 pretreated groups, the change aroused by LIPUS was markedly diminished (Figure S4G, Supporting Information). In brief, LIPUS promotes extracellular matrix anabolic partially via chondrocyte autophagy.

### 2.4 The Mechanosensitive Ion Channel TRPV4 is Pivotal for Mediating Calcium Activation and the Subsequent Promotion of Autophagy Induced by LIPUS

**Figure 5:**
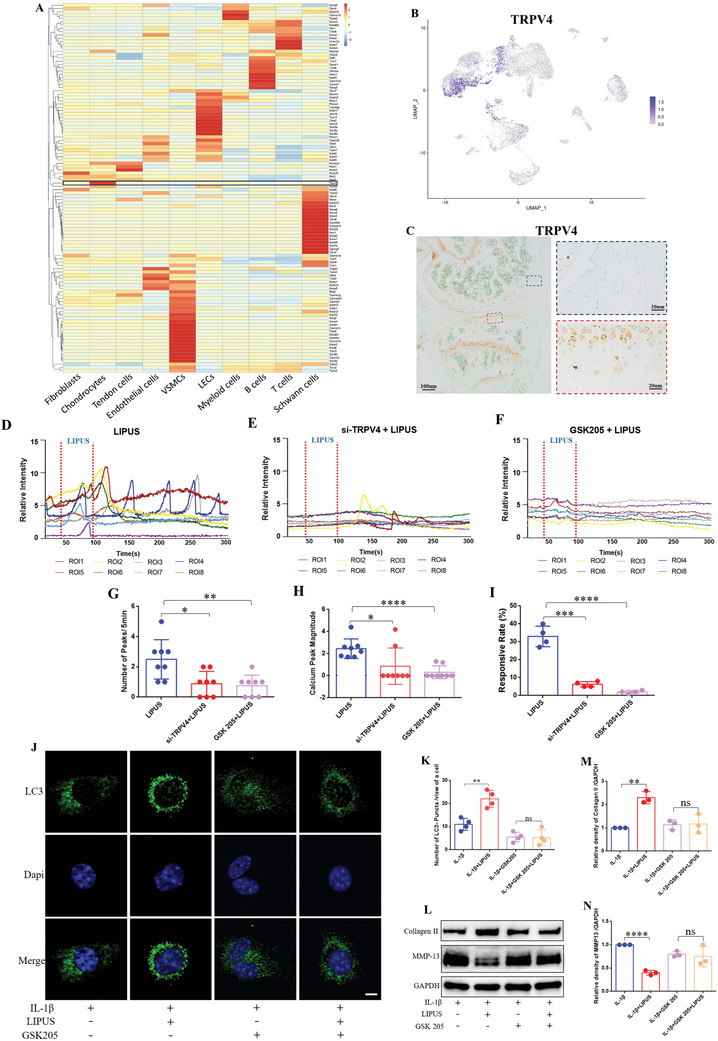
TRPV4 mediated Ca^2+^ signaling participates the regulation of LIPUS to inflammatory chondrocyte. A) Heatmap showing ion channel gene expression in all cells from the mouse ACLR model of PTOA. B) The express distribution of TRPV4 in UMAP plots. C) Immunohistochemical staining of TRPV4 in the mouse knee joint. Calcium transient relative fluorescence intensity of ROIs with LIPUS stimuli in control group D), in TRPV4‐knockdown group E), and in the TRPV4‐blocked group F). Quantification of the number G) (*n* = 8), the magnitude H) (*n* = 8) of calcium peaks, and the responsive rate of cells I) (*n* = 4). J) Immunofluorescence was used to detect the LC3‐puncta after the chondrocytes was co‐incubated with GSK205. Scale bar, 5 µm. K) Quantification of LC3‐puncta in chondrocytes (*n* = 4). WB L) and densitometry analysis of Collagen II M) and MMP‐13 N) protein expression of chondrocyte co‐incubated with GSK205 following LIPUS treatment (*n* = 3). Data are presented as means ± SD. Statistical analysis was performed using Student's t test. ****(*P* < 0.0001), ***(*P* < 0.001), **(*P* < 0.01), *(*P* < 0.05), ns (0.05 < P).

Chondrocytes exhibit intrinsic mechanosensitivity owing to the various expression of mechanically‐activated (MA) ion channels, such as Piezo1, Piezo2, and TRP family channels.^[^ ^17^, ^18^, ^19^, ^20^, ^59^ ^]^ In order to further explore the mechanotransduction between LIPUS and chondrocytes, we obtained the published scRNA‐seq datasets (GSE211584: arthritis specimens from mice subjected to the ACLR model of PTOA)^[^ ^60^, ^61^ ^]^ from NCBI Gene Expression Omnibus for integration and subsequent analysis. Based on the marker genes collected in the public database CellMarker 2.0,^[^ ^62^ ^]^ cells were reclassified into 10 distinct clusters comprising fibroblasts, chondrocytes, tendon cells, endothelial cells, vascular smooth muscle cells, lymphatic endothelial cells, myeloid cells, B cells, T cells, and Schwann cells (Figure S5A,B, Supporting Information). We further screened the relative expression of more than 200 mouse ion channel‐related genes from NCBI in the above 10 clusters, excluded the genes with very low expression, and plotted the expression heat maps of the remaining 112 genes using pheatmap (version 1.0.12) (**Figure** **5**A; and Table S1, Supporting Information). We found that most of the genes were concentrated and highly expressed in specific cells. The TRPV4 gene caught our attention since it was specifically and highly expressed in chondrocytes. We further verified the express distribution of TRPV4 in uniform manifold approximation and projection (UMAP) plots (Figure 5B) and by immunohistochemistry of the mouse joint (Figure 5C), confirming the mechanosensitive ion channel gene TRPV4 abundantly expressed in mouse chondrocytes and specifically.

TRPV4 has been shown to participate chondrocyte differentiation and OA progression.^[^ ^29^, ^30^, ^63^ ^]^ We speculated that TRPV4 may be a pivotal integrator between extracellular physicochemical stimuli and intracellular biochemical signaling of chondrocyte. Therefore, we established a TRPV4 knock down (TRPV4‐KD) model by siRNA, the knockout efficiency was verified by qPCR (Figure S6A, Supporting Information) and WB (Figure S6B,C, Supporting Information), proving the chondrocyte TRPV4‐KD model was successfully established. We conducted calcium imaging on TRPV4‐KD chondrocytes and those pretreated with GSK205, a specific TRPV4 blocker,^[^ ^64^ ^]^ during real‐time LIPUS treatment, following the same detailed imaging procedure as previously utilized. Time‐lapse images of chondrocytes across the 3 groups are displayed in (Figure S7A and Movies S6–S8, Supporting Information). Eight cells marked by circles with different colors were randomly selected to show calcium oscillations in each group. Each curve color‐matched to the corresponding cell, depicted the calcium transient of an individual chondrocyte (Figure 5D–F). The calcium oscillation in the control group (LIPUS alone group) is markedly more active, whereas the curves in the other two groups were comparatively flat, which showed very few and lower magnitude calcium peaks. And statistical data of the number (Figure 5G), magnitude (Figure 5H) of calcium peaks and the responsive rate of cells (Figure 5I) induced by LIPUS were both inhibited when TRPV4 was knocked down or blocked. The results demonstrated that the mechanosensitive ion channel TRPV4 mediates the regulation of calcium signaling in chondrocytes by LIPUS.

To investigate whether TRPV4 mediate LIPUS upregulation of chondrocyte autophagy, we pretreated the chondrocytes with GSK205 prior to LIPUS. The protein lever of LC3 II (Figure S7B,C, Supporting Information), number of LC3‐Puncta (Figure 5J,K), and the number of autophagosomes (Figure S7D, Supporting Information) up‐regulated by LIPUS were both crippled while blocking TRPV4. Furthermore, we hypothesized that TRPV4 is also involved in the cartilage homeostasis maintaining by LIPUS. WB as shown in (Figure 5L–N), there was no significant difference of Collagen II or MMP‐13 in GSK205 pretreated cells between IL‐1β and IL‐1β+LIPUS groups. Immunofluorescence shown a consistent result (Figure S7E–G, Supporting Information). In brief, LIPUS maintaining cartilage homeostasis also partially depends on TRPV4.

### 2.5 Low dosage of Adenophostin A Enhances LIPUS‐Induced Calcium Activation and its Bioprotective Effect on Chondrocyte

**Figure 6:**
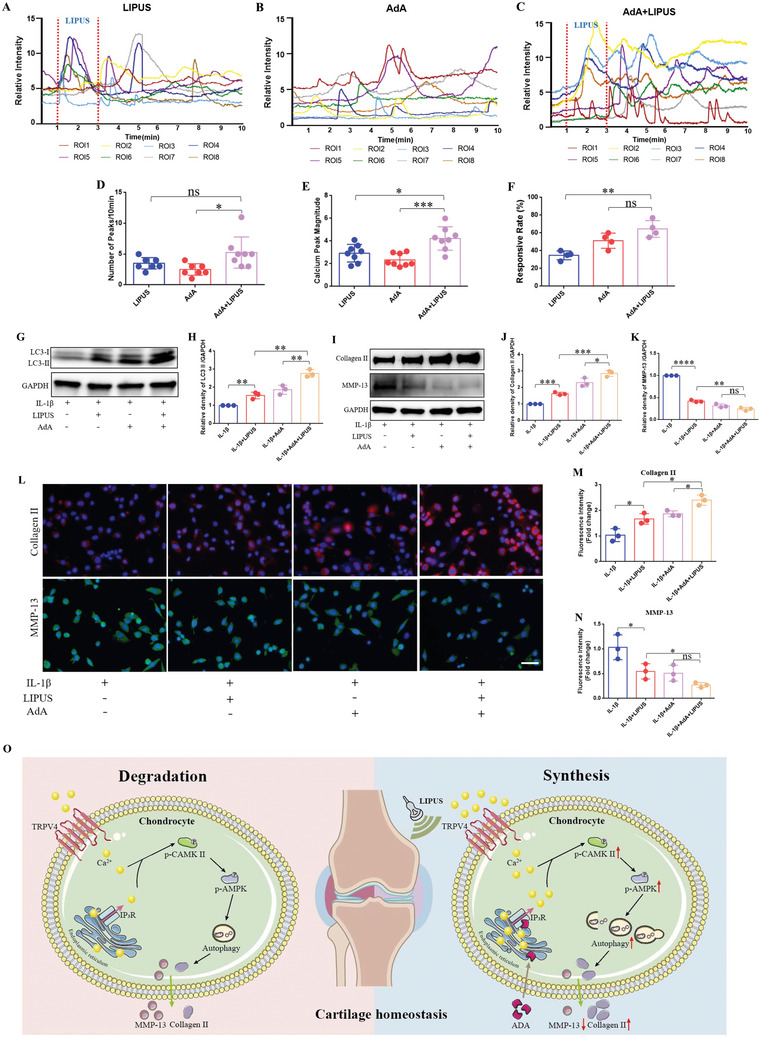
The intracellular calcium agonist AdA has a synergistic effect with LIPUS in regulating inflammatory chondrocyte. Calcium transient relative fluorescence intensity of ROIs in LIPUS alone A), in AdA alone B), in the combination of LIPUS and AdA C). Quantification of the number D) (*n* = 8), the magnitude E) (*n* = 8) of calcium peaks, and the responsive rate of cells F) (*n* = 4). WB G) and densitometry analysis of LC3 H) protein expression of chondrocyte co‐incubated with AdA following LIPUS treatment (*n* = 3). WB I) and densitometry analysis of Collagen II J) and MMP‐13 K) protein expression of chondrocyte co‐incubated with AdA following LIPUS treatment (*n* = 3). L) Immunofluorescence was used to detect the expression of Collagen II and MMP‐13 after the chondrocytes was co‐incubated with AdA. Scale bar, 20µm. Quantification the relative fluorescent intensity of Collagen II M) and MMP‐13 N) in chondrocytes (*n* = 3). O) Schematic diagram of LIPUS alleviates OA via calcium signaling‐dependent autophagy of chondrocyte. Data are presented as means ± SD. Statistical analysis was performed using Student's t test. ****(*P* < 0.0001), ***(*P* < 0.001), **(*P* < 0.01), *(*P* < 0.05), ns (0.05 < P).

Studies have demonstrated that extracellular Ca^2+^ is essential for initiating spontaneous ([Ca^2+^]_i_) in chondrocytes, and the release of Ca^2+^ stored in the endoplasmic reticulum （ER） into the cytosol is also essential for calcium oscillation, in which the phospholipase C‐Inositol 1,4,5‐trisphosphate (PLC‐IP3) pathway plays an important role in the start‐up of spontaneously [Ca^2+^]_i_.^[^ ^39^ ^]^ We attempted to find a combination therapy strategy based on the mechanism studied above. We utilized the exogenous Inositol 1,4,5‐trisphosphate receptor (IP3R)‐specific agonist Adenophostin A (AdA)^[^ ^65^ ^]^ in conjunction with LIPUS to observe calcium signaling across the groups as previous. Time‐lapse images of chondrocytes in 3 groups are shown in (Figure S8 and Movies S9–S11, Supporting Information). 8 cells circled with different colors were randomly selected to illustrate calcium oscillations in each group. Each curve of the corresponding color depicted the calcium transient of an individual chondrocyte (**Figure** **6**A–C). Our findings indicated that calcium oscillations in the LIPUS+AdA group were significantly more pronounced than with LIPUS or AdA alone. The statistical analysis revealed a greater number of calcium peaks (Figure 6D), enlarged magnitude of calcium peaks (Figure 6E), and elevated response rate (Figure 6F) in LIPUS+AdA group, which confirms a synergistic effect of AdA with LIPUS in activating calcium signaling of chondrocytes.

On the other hand, we validated the synergistic effect of LIPUS in combination with the intracellular calcium agonist AdA at the protein level. WB results showed that LIPUS up‐regulated the expression of LC3 II in chondrocytes, so as the low‐dose AdA alone, while this function was much strengthened when combined them together (Figure 6G,H). We further focused on the expression of Collagen II and MMP‐13 (Figure 6I–K), consistent with the immunofluorescence results (Figure 6L–N), showed that the combing of LIPUS with low‐dose AdA further increased the expression of Collagen II, and decreased the expression of MMP‐13. Indicating that the combination of LIPUS with low‐dose AdA further enhanced its anabolic effect on the ECM. This suggests a novel therapeutic strategy for the combined use of LIPUS in OA treatment.

## 3 Discussion

With the aging of the population, the substantial burden of osteoarthritis on society has garnered increasing attention.^[^ ^6^ ^]^ It is widely acknowledged that biomechanical factors are crucial for articular cartilage development and homeostasis.^[^ ^15^, ^66^ ^]^ As a mechanosensitive cell type, chondrocytes detect changes in the mechanical microenvironment and regulate key biological processes including cell proliferation, migration, differentiation, and apoptosis, processes integral to cartilage development and post‐injury homeostasis.^[^ ^67^ ^]^ Therefore, a deeper understanding of chondrocytes' sensitivity to the mechanical microenvironment and its role in maintaining cartilage homeostasis is essential for finding new strategies for OA treatment.

LIPUS is a non‐invasive and safe physical therapy, which can influence specific signaling pathways and regulate the cellular functions through the mechanical effects of acoustic wave.^[^ ^9^, ^68^ ^]^ Ca^2+^, a universal second messenger in organisms, is one of the earliest responses of chondrocytes to physical stimuli.^[^ ^15^, ^21^, ^24^ ^]^ Ultrasound‐induced mechanical stimulation is known to cause transient increases in intracellular Ca^2+^ levels in various cell types, such as ovarian cells,^[^ ^69^ ^]^ MC3T3‐E1 cells,^[^ ^70^ ^]^ and osteocytes.^[^ ^71^ ^]^ In this study, we explored the Ca^2+^ signal as a bridge between LIPUS and chondrocytes. We captured real‐time calcium oscillations in chondrocytes induced by LIPUS, surpassing previous reports by providing direct evidence for the dynamic “visualization” of mechanical transduction between LIPUS and chondrocytes, thereby establishing a foundation for future research. Calcium oscillatory signals can be transmitted not only from cell to cell,^[^ ^72^ ^]^ but also amplified by calcium‐induced calcium release (CICR), travels across the cytosol in the form of waves.^[^ ^73^ ^]^ We found that the calcium oscillation of primary chondrocytes was rapidly enhanced during LIPUS treatment, and it was weakened after LIPUS treatment, but there were occasional peaks appeared delay in vitro. And in vivo, the calcium oscillation of chondrocytes was not only enhanced during LIPUS treatment, but also showed a continuous or even strengthen after the treatment. We hypothesized that the LIPUS‐treated may lead to the continuous enhancement of chondrocyte calcium oscillation through cell‐cell interactions, CICR, or any other cascade signal amplification caused by calcium signaling itself. Which further suggests that chondrocytes have a unique spatiotemporal pattern in response to LIPUS stimulation, but the specific mechanism still needs more investigation.

Autophagy, a highly conserved cellular degradation and metabolic pathway in eukaryotes, enables cells to maintain biological homeostasis through degrading their own components.^[^ ^33^, ^34^ ^]^ Chondrocytes are encapsulated in their own secreted ECM, sensitive to nutrients due to the unique hypoxic and nutrient‐poor environment, therefore, autophagy is crucial for maintaining chondrocyte homeostasis and normal physiological function.^[^ ^34^, ^74^ ^]^ Targeting chondrocyte autophagy may offer a promising therapeutic strategy for OA treatment. In our study, we demonstrated the up‐regulation of LIPUS on chondrocyte autophagy both in vivo and in vitro. These findings align with a recent study showing that LIPUS reduces YAP expression by restoring impaired autophagy and inhibiting the YAP‐RIPK1 interaction, thus delaying osteoarthritis progression.^[^ ^41^ ^]^ Furthermore, leveraging live imaging technology, we obtained significantly improved results, visualizing the dynamic process of LIPUS‐induced autophagic flux in chondrocytes. Moreover, experiments under autophagy‐deficient conditions confirmed that LIPUS‐mediated alleviation of OA progression is dependent on chondrocyte autophagy. In a newly research, which provides a conceptual therapeutic solution for the prevention of osteoarthritis via alleviation of lysosomal destabilization in chondrocyte.^[^ ^75^ ^]^ However, our study differs from previous research in that it places a greater emphasis on exploring the mechanotransduction between LIPUS and chondrocytes.

As a ubiquitous intracellular second messenger, Ca^2+^ plays a key role in regulating numerous biological processes, including autophagy.^[^ ^26^, ^27^, ^28^ ^]^ Oxoglaucine mediates Ca^2+^ influx through TRPV5/CaMKII signaling, thereby promoting chondrocyte autophagy to alleviate OA progression.^[^ ^76^ ^]^ Subsequent study has shown that autophagy induces Ca^2+^ transients and oscillations on the ER membrane's outer surface, leading to phase separation of FIP200 and specifying autophagosome initiation sites on the ER.^[^ ^77^ ^]^ This finding provides compelling evidence for the interplay between calcium signaling and autophagy. Additionally, our study reveals that LIPUS upregulates autophagy through a calcium‐dependent signaling pathway CaMK II‐AMPK, revealing a previously unrecognized mechanism of LIPUS.

We sought to further elucidate the specific mechanisms through which LIPUS influences calcium signaling of chondrocytes. It is widely recognized that chondrocytes possess a range of mechanically activated ion channels on their surface, which convert physical stimuli into intracellular signaling cascades.^[^ ^17^, ^19^, ^20^ ^]^ We obtained the published scRNA‐seq datasets (GSE211584)^[^ ^60^, ^61^ ^]^ from GEO and reclassified cells into 10 clusters including chondrocytes. We identified the ion channel TRPV4, which is preferentially expressed in chondrocytes. In line with previous studies, TRPV4 plays a critical role in the development of chondrogenesis and osteoarthritis. Detailly, TRPV4 is a mechanosensitive, non‐selective Ca^2+^ ion channel that can be activated by a variety of physical and chemical stimuli, such as osmotic pressure, mechanical stress, temperature changes, and chemical agents.^[^ ^78^ ^]^ Furthermore, it is a promising target for skeletal regeneration, as it mediates chondrocyte proliferation and matrix synthesis in response to mechanical loading during skeletal development.^[^ ^79^ ^]^ Additionally, TRPV4 is implicated in the chondrocyte response to dynamic loading, influencing ECM synthesis, and its deficiency can expedite the progression of OA.^[^ ^67^ ^]^ A recent study was consistent well with our finding demonstrated that LIPUS modulates TRPV4 channels in primary cilia.^[^ ^80^ ^]^ However, the distribution of TRPV4 is not limited to primary cilia, and the underlying mechanism remains incompletely understood. Our data further indicate that TRPV4‐mediated calcium signaling is essential for LIPUS regulation of chondrocytes.

Ultimately, we aimed to develop a combination therapy strategy based on the aforementioned mechanisms. Studies have shown that the release of Ca^2+^ from the ER into the cytosol is a primary source of calcium oscillations, with the IP3 receptor (IP3R) being the principal channel participating this release.^[^ ^81^ ^]^ Research indicates that TRPV4‐mediated Ca^2+^ influx, upon binding to IP3 in endothelial cells, further activates IP3R, leading to increased Ca^2+^ release into the cytoplasm and inducing a more extensive and sustained calcium wave that enhances vascular tone regulation.^[^ ^82^ ^]^ We utilized the exogenous IP3R‐specific agonist AdA^[^ ^65^ ^]^ in conjunction with LIPUS for further investigation. The intracellular calcium agonist AdA exhibits a synergistic effect when combined with LIPUS in modulating inflammatory chondrocytes.

These results highlight intriguing discrepancies in the quantified metrics—number of calcium peaks, amplitude, and response rate, which warrant further discussion. Upon comparison of the combination group with the AdA‐only group, LIPUS increased the number and amplitude of calcium peaks but did not increase the calcium response rate. AdA increased the amplitude and response rate of calcium peaks, yet only showed a non‐significant increasing trend in the number of peaks. This indicates that LIPUS and AdA prioritize distinct aspects of calcium signaling activation. Specifically, LIPUS increases the frequency and amplitude of oscillations in individual chondrocytes, whereas AdA enhances the cellular calcium response rate. The [Ca^2+^]i peak fluctuations from a single chondrocyte exhibit a unique spatiotemporal pattern that varies among cells, serving as a distinctive “fingerprint”.^[^ ^39^ ^]^ Nevertheless, the specific waveforms of calcium oscillations and the biological implications of varied calcium activation modes remain largely enigmatic, with few reports on decoding these oscillations. This result offers a novel possibility of strategy combining with LIPUS for the treatment of OA.

We didn't verify the combination therapeutic in animal experiment due to the intra‐articular injection is invasive which may cause additional damage to the joints. In addition, the targeting of chondrocytes by AdA in vivo is not yet clear. Wrapping AdA in chondrocyte‐targeting material, combined with LIPUS, may be the future direction of us. In the study, we did not evaluate the dose‐response relationship of LIPUS. Despite conducting exploratory experiments with non‐statistically distinct results, the dose‐response relationship of LIPUS merits attention. There is a scarcity of clinical trials on LIPUS for OA treatment, but the intensity and effects are different.^[^ ^9^, ^10^, ^11^ ^]^ The sonodynamic action is impacted by the combination of frequency, intensity, duty cycle, and ultrasound application time. Variations in cell culture experimental setups, such as transducer position, cell‐transducer distance, coupling medium thickness, and culture type, also affect the sonodynamic response.^[^ ^83^ ^]^ Therefore, a meticulously designed and rigorously controlled experimental framework is essential for ultrasound‐related research, with the need for further optimization of specific regulatory parameters.

In conclusion, our study has demonstrated that LIPUS activates calcium signaling in chondrocytes and alleviates OA partially by promoting chondrocyte autophagy in a calcium‐dependent manner. Furthermore, the mechanosensitive ion channel TRPV4 mediates the regulatory effects of LIPUS on chondrocytes. Lastly, we identified a potential combination therapy strategy based on the aforementioned mechanisms, offering novel insights into the treatment of OA through biomechanical intervention (Figure 6O).

## 4 Experimental Section

### Isolation and Culture of Mouse Primary Chondrocytes

Primary chondrocytes were isolated from the knee of new born less than 5‐day‐old mice. Knee joints were first digested by 0.25% trypsinase (Gibco/Life Technologies, USA) at 37 °C for 15 min. Culled out adjacent muscles, ligaments and bone tissues under the stereomicroscope (Olympus BX51, Japan) observation to obtained the articular cartilage. Chondrocytes were isolated from the cartilage by additional digestion with 0.1% collagenase Π (Gibco/Life Technologies, USA) overnight at 37 °C in a 5% CO_2_ incubator as described in Gosset et al.^[^ ^84^ ^]^ Cells were seeded in 35‐mm‐diameter dishes or 12‐well plates, cultured by Dulbecco's modified Eagle's medium/ F12 (1: 1) contained 1% penicillin/streptomycin (HyClone, USA) and 10% FBS (Gibco/Life Technologies, USA) and incubated in the environment as before. The culture medium was changed once every other day. The high‐purity chondrocytes were obtained is shown in Figure 3F.

### Fluorescence Imaging of Calcium Oscillation

To evaluate the changes in intracellular calcium concentrations, a calcium‐sensitive fluorescence indicator, Fluo4 AM (Ex = 494 nm, Em = 516 nm, Beyotime, Shanghai, China), was used to label the cells according to the manufacturer's instructions. Briefly, confluent 35‐mm glass‐bottom microwell dishes containing cells were first washed twice in HBSS without Ca^2+^ and then labled with 4µM Fluo‐4 AM diluted by HBSS without Ca^2+^ at 37 °C and 5% CO_2_ for 20–30 min (adjust according to the state of cells appropriately). The cell dishes were shielded from light, and then washed 3 times at room temperature by Ca^2+^‐HBSS. Next, the cells were visualized and imaged to observe calcium oscillations using an Olympus IX81 fluorescence microscope (Olympus Optical, Japan) (Figure S1A, Supporting Information). Time‐lapse sequences were collected every 0.5 s. Eight cells marked by circles with different colors were randomly selected to show calcium oscillations in each group. Each curve of the corresponding color represented the calcium transient relative fluorescence intensity of the single cell. Between the two red lines in the calcium oscillation curve represents the LIPUS treatment (parameters: ultrasound frequency 1.5 MHz; pulse repetition frequency: 1.0 KHz; intensity 30 mW cm^−2^; duty cycle 20%) period.

Images were analyzed using individual circular regions of interest (ROI) on ImageJ. The amplitude of signals was presented as relative fluorescence changes (ΔF/F) after background subtraction.^[^ ^85^ ^]^ A calcium peak was regarded when a transient increase of [Ca^2+^]_i_ was 1.25 times above the baseline intensity of each cell. The total number of peaks during recording in each cell was counted. When one calcium peak was appeared during recording, this cell was regarded as a responsive cell. The percentage of responsive cells was quantified by the number of total cells in the field of view.^[^ ^86^ ^]^ The calcium peak magnitude was reported as the mean fold increment of the peak intensity over the mean baseline fluorescence intensity in ROI.^[^ ^24^ ^]^

### Alcian Blue Staining of Zebrafish

Animal experiments were approved by Ethics Committee of the First Affiliated Hospital of Chongqing Medical University (No: 2022‐K240). Acid‐free protocols were adapted to perform Alcian blue staining of cartilage structures. At 5dpf, euthanize the larvae with ice‐water mixture, fixed in 4% PFA overnight at room temperature. The next day, rinsed them several times with PBST. Cartilage was stained 1 h in 10 mm MgCl_2_, 80% EtOH and 0.04% Alcian blue (Sigma‐Aldrich, Germany). The larvae were washed by different concentrations of ethanol (75%, 50%, and 25%) in turn to remove excess staining. Pigmentation was bleached in a H_2_O_2_ solution (H_2_O_2_ 3%, KOH 0.5%) and finally the larvae were rinsed in turn by solution of 25% glycerol, 0.1% KOH and 50% glycerol, 0.1% KOH, stored in this solution at 4 °C or take photos by stereo microscope finally.^[^ ^42^ ^]^

### Calcium Imaging of Zebrafish Juveniles

The GCaMp6s fish line used in this study was obtained from the Zebrafish Resource Center of China. All zebrafish were housed in semi‐closed recirculation housing systems and kept at a constant temperature (28.5 °C) on a 14:10 h light: dark photoperiod. Zebrafish larvae at 5dpf were anesthetized by 0.2% Tricaine (Sigma‐Aldrich, Germany) and then immobilized upon a confocal dish bottom with low‐melting‐point agarose. Placed the LIPUS probe into the dish, used egg water as the propagation medium, and took care to avoid air bubbles (Figure 2B,C). The living larvae were imaged every second with the Olympus confocal microscope (Olympus, Japan) (Figure S1B,C, Supporting Information). Five cells marked by circles with different colors were selected to show calcium oscillations. Each curve of the corresponding color represented the calcium transient relative fluorescence intensity of the single cell. Between the two red lines in the calcium oscillation curve represents the LIPUS treatment (parameters: ultrasound frequency 1.5 MHz; pulse repetition frequency: 1.0 KHz; intensity 30 mW cm^−2^; duty cycle 20%) period.

### Destabilized Medial Meniscus (DMM)‐Induced OA

Animal experiments were approved by Ethics Committee of the First Affiliated Hospital of Chongqing Medical University (No: 2022‐K240). Eight weeks old C57BL/6 male mice were purchased from (Beijing Vital River Laboratory Animal Technology, China). Animals were housed in a specific pathogen‐free vivarium with a 12h/12h light/dark cycle. Rodent chow and water were provided ad libitum. After a week of acclimatization, DMM surgery was performed on the right knee joints of the 9‐week‐old male mice according to the procedure described in a previous study.^[^ ^47^ ^]^ Briefly, medial meniscus of the right knee was removed using aseptic surgical procedures. As a control, sham operation was performed on the left knee joint with medial capsulotomy only. At the end of the experiment, the mice were euthanatized using an overdose of anesthetics. Knee joints were dissected for histological examination.

### Mouses and Cells Treated with LIPUS

Two days after DMM surgery, the mice were anesthetized and smear coping gel on the right knee evenly after preparing skin (≈1 mm thick). The LIPUS device (TSZ‐100; TOPSONIC, China) was set below the injured knee for 20 min/d, 5 d/week, for 2 weeks at the corresponding parameter. The LIPUS device was affixed to the control mice without turning‐on at the same time.^[^ ^45^ ^]^

Chondrocytes cultured in 35‐mm‐diameter dishes were treated with LIPUS (TSZ‐100; TOPSONIC, China) at the corresponding parameter. The stimulations were executed in a sterile environment at room temperature. The acoustic gel (≈1 mm thick) was evenly covered as a medium between the transducer and cell plate to ensure optimal ultrasound exposure.^[^ ^8^ ^]^ Meanwhile, the control group was put in the identical environment with sham stimulation.

### Immunohistochemistry (IHC)

The knee joint sections were dewaxed with xylene and subjected to trypsin ‐mediated antigen retrieval before the sections were sealed with goat serum. After incubation with the primary antibody overnight, the secondary antibody and horseradish peroxidase‐labeled streptavidin biotin were added. Sections were dyed with methyl green. Primary antibodies: LC3 (1:100; Sigma‐Aldrich, L7543), ATG7 (1:100; Proteintech, 10088‐2‐AP), Collagen II (1:100; Chondrex, 7005), MMP‐13(1:100; Proteintech, 18165‐1‐AP), TRPV4(1:100; Abcam, ab39260).

### Western Blotting (WB)

Chondrocytes were collected with RIPA lysis buffer (Beyotime, China) containing protease inhibitors for protein extraction. Proteins were separated by 12% SDS‐PAGE and transferred to a PVDF membrane (Millipore, USA). After blocking with 5% nonfat milk in TBST buffer for 1 h, the membrane was incubated with specific primary antibodies at 4 °C overnight. The membranes were washed three times with TBST, and incubated with secondary antibodies anti corresponding species at 37 °C for 1 h. Before immunoreactivity was detected by chemiluminescence, the membranes were washed three times with TBST. At last, the signal was detected using ChemiScope 6000 (Clinx Science Instruments, China). Primary antibodies including: LC3 (1:1000; Sigma‐Aldrich, L7543), ATG7 (1:1000; Proteintech, 10088‐2‐AP), Collagen II (1:1000; Chondrex, 7005), MMP‐13 (1:1000; Proteintech, 18165‐1‐AP), TRPV4(1:1000; Abcam, ab39260), GAPDH (1:1000; Proteintech, 60004‐1), CAMK II (1:1000; Abcam, ab181052), pCAMK II (1:1000; CST, 12 716), AMPK (1:1000; CST, 2532), and pAMPK (1:1000; CST, 2535).

### Transmission Electron Microscopy

Briefly, chondrocytes after corresponding treatment were fixed in 2% glutaraldehyde overnight at 4 °C. Prepared ultrathin sections (60–80 nm) and then mounted on copper grids. Autophagosomes were detected with an electron microscope (JEM‐1011, Olympus, Japan).

### Immunofluorescence (IF)

Chondrocytes were seeded into 35 mm glass bottom confocal culture dish and treated as before. Fixed the samples by 4% PFA for 10 min after corresponding treatment. Then, blocked the chondrocytes with goat serum for 1 h. Next, incubated chondrocytes with primary antibodies at 4 °C overnight. After wash out the primary antibodies by PBS, corresponding second antibodies were incubated. Stained the nucleus with DAPI for 5 min. Finally, the images were captured by a laser scanning confocal microscope (Olympus, Japan). Primary antibodies including: LC3 (1:100; Sigma‐Aldrich, L7543), iNOS (1:100; Proteintech, 18985‐1‐AP), Collagen II (1:100; Chondrex, 7005), MMP‐13 (1:100; Proteintech, 18165‐1‐AP).

### Acquisition of Transgenic Mice

Animal experiments were approved by Ethics Committee of the First Affiliated Hospital of Chongqing Medical University (No: 2022‐K240). The Atg7^flox/flox^ mouse line was a generous gift from Dr. Liu Cao (Institute of Translational Medicine, China Medical University), and Col2a1‐CreER^T2^ mice line was a generous gift from Dr. Chuxia Deng (Faculty of Health Sciences, University of Macau). All mice were kept on the C57BL/6J background and maintained in a standard, specific‐pathogen‐free facility with a 12h/12h light/dark cycle. Atg7^flox/flox^; Col2a1‐CreER^T2^ mice were designated ATG cKO and control littermates (Atg7^flox/flox^) were referred to as controls. For postnatal activation of CreER^T2^, eight‐week‐old male mice were injected intraperitoneally with 100 mg kg^−1^ tamoxifen (T5648‐5G, Sigma‐Aldrich, German) in corn oil (C8267, Sigma‐Aldrich, German) once a day for 5 consecutive days.^[^ ^87^ ^]^ nine‐week‐old male transgenic mice were used for the experimental OA induced by DMM.

### Histological Analysis

Mouse knee joints were fixed in 4% paraformaldehyde for 24 h, then decalcified in 10% EDTA (pH 7.4) for 14 days. The tissues were embedded in paraffin and sectioned continuously (5 µm thick) and serial sections were obtained from the medial and lateral compartments at 50 µm intervals. Seven to eight representative mid‐sagittal sections were selected and deparaffinized in xylene, then hydrated with graded ethanol. Cartilage destruction was detected using Safranin‐O/fast green or HE staining. Slides were imaged using an Olympus‐BX43 microscope (Olympus) with CellSens (v4.1) software. Degeneration of articular cartilage was quantified by three observers using the OARSI scoring system under blinded conditions.^[^ ^2^ ^]^ To measure the thicknesses of articular cartilage, the mean of three measurements (locations as indicated in Figure S2F, Supporting Information by blue lines) from 6 consecutive sections in the middle of the joint (total of 18 measurements). The final measurement is an average of these sections.^[^ ^88^ ^]^

### Single‐Cell RNA Sequencing (scRNA‐seq)

The published scRNA‐seq datasets (GSE211584: arthritis specimens from mice subjected to the ACLR model of PTOA)^[^ ^60^, ^61^ ^]^ were obtained from NCBI Gene Expression Omnibus for integration and further analysis. Based on the marker genes collected in the public database CellMarker 2.0,^[^ ^62^ ^]^ cells were reclassified into 10 clusters. Further the relative expression of more than 200 mouse ion channel‐related genes were screened from NCBI in clusters, excluded the genes with very low expression, and plotted the expression heat maps of the remaining 112 genes by pheatmap (version 1.0.12). It was found that most of the genes were concentrated and highly expressed in specific cells, while the TRPV4 gene was more specifically expressed in chondrocytes. Further the expression distribution of TRPV4 was verified in UMAP plots.

### Transfection

To observe autophagic flux, chondrocytes were infected with dual‐tagged LC3 (mRFP‐GFP‐LC3) adenovirus (Hanbio Biotechnology, Shanghai, China). In brief, the cells were incubated by 100 MOI lentivirus for 24 h and treated with IL‐Iβ for 24 h. Then, the cells were treated with or without LIPUS treatment. After 2 h, the cells were harvested. The images were captured by a laser scanning confocal microscope (Olympus, Japan).

To knockdown TRPV4, scramble small‐interfering RNA (siRNA) and TRPV4 siRNA were constructed by RiboBio (Guangzhou, China). Lipofection was used to deliver 100 nmol L^−1^ TRPV4 siRNA into chondrocyte by Lipofectamine 3000 (Invitrogen, USA) following the manufacturer's instructions. 48 h after transfection, the silencing efficiency was determined by qRT‐PCR and western blotting.

### Toluidine Blue Staining

To observe the proteoglycan side chains in mature aggrecan (AGC), the primary chondrocytes in the wells were washed with PBS and fixed with 4% paraformaldehyde at room temperature for 10 min. Chondrocytes were washed twice with PBS again and stained with toluidine blue for 15 min, after wash out the dye with PBS, the cells were observed and photographed under a microscope.

### Real‐Time Polymerase Chain Reaction

Total RNA was isolated from chondrocytes using the TRIzol reagent (Invitrogen, USA) and used to generate cDNA template for qPCR, which was carried out on the Mx3000P system (Takara Bio, Japan) using the SYBR Green RT‐PCR Kit (Takara Bio, Japan). All samples were measured in triplicate. The forward primer sequence of TRPV4 was 3′‐ATGGCAGATCCTGGTGATGG‐5′ and the reverse primer sequence was 3′‐GGAACTTCATACGCAGGTTTGG‐5′.

### Statistical Analysis

Each experiment was conducted independently at least three times. Data were presented as means ± SD and analyzed using two‐tailed Student's t test or oneway analysis of variance (ANOVA). Statistical analysis was performed using GraphPad Prism 8.0 software. *p* < 0.05 was considered statistically significant, while ns indicates that there is no statistical difference (*p < 0.05, **p < 0.01, and ***p < 0.001).

## Conflict of Interest

The authors declare no conflict of interest.

## Author Contributions

M.T.G., X.Y.H., L.B., and W.H. contributed equally to this work and are the first co‐authors. All authors were involved in drafting the article or revising it critically for important intellectual content, and all authors approved the final version to be published. M.T.G., Y.Z., and D.Q.B. performed the study conception and designed the study. M.T.G., B.L., W.H., X.Q.P., Y.T., G.Y.X., X.H.L., and L.K. acquired data. M.T.G., X.Y.H., L.B., and B.Z. performed the interpretation and analysis of data. M.T.G., X.Y.H., L.B., and L.K. wrote the manuscript. D.Q.B., Y.Z., L.K., and L.C. applied the funding acquisition and project administration.

## Supporting information



Supporting Information

Supplemental Table 1

Supplemental Movie 1

Supplemental Movie 2

Supplemental Movie 3

Supplemental Movie 4

Supplemental Movie 5

Supplemental Movie 6

Supplemental Movie 7

Supplemental Movie 8

Supplemental Movie 9

Supplemental Movie 10

Supplemental Movie 11

## Data Availability

The data that support the findings of this study are available from the corresponding author upon reasonable request.
