# Supplementary material for: LIPUS Promotes Calcium Oscillation and Enhances Calcium Dependent Autophagy of Chondrocytes to Alleviate Osteoarthritis
Source: Adv Sci (Weinh). 2025 Feb 27;12(16):2413930. doi: 10.1002/advs.202413930 (PMC12021083; doi:10.1002/advs.202413930)
Supplement: Supplementary file 1 — Supporting Information [file ADVS-12-2413930-s004.docx]

Supporting Information

Title: LIPUS promotes calcium oscillation and enhances calcium dependent autophagy of chondrocytes to alleviate osteoarthritis

*Mengtong Guan, Xiaoyu Han, Bo Liao, Wang Han, Lin Chen, Bin Zhang, Xiuqin Peng, Yu Tian, Gongyi Xiao, Xinhe Li, Liang Kuang*, Ying Zhu*, Dingqun Bai**

M.T. Guan, X.Y. Han, L. Bo, W. Han, X.H. Li, Y. Zhu, D.Q. Bai

Department of Rehabilitation Medicine, Key Laboratory of Physical Medicine and Precision Rehabilitation of Chongqing Municipal Health Commission. The First Affiliated Hospital of Chongqing Medical University, Chongqing, 400010, China.

E-mail: yingzhu517@hospital.cqmu.edu.cn (Y. Zhu)

E-mail: baidingqun@hospital.cqmu.edu.cn (D.Q. Bai)

L. Chen, B. Zhang, X.Q. Peng, Y. Tian, L. Kuang
Center of Bone Metabolism and repair laboratory for Prevention and rehabilitation of Training injuries State Key laboratory of Trauma Burns and combined injury Trauma center Research Institute of Surgery Daping Hospital Army Medical University (Third Military Medical University), Chongqing, 400000, China.

E-mail: kliang1989@tmmu.edu.cn (L. Kuang)

X.Y. Han, X.H. Li, D.Q. Bai

State Key Laboratory of Ultrasound in Medicine and Engineering, Chongqing Medical University, Chongqing, 400016, China.

E-mail: baidingqun@hospital.cqmu.edu.cn (D.Q. Bai)

G.Y. Xiao

Department of Orthopedics, Chonggang General Hospital, Chongqing, 400000, China.


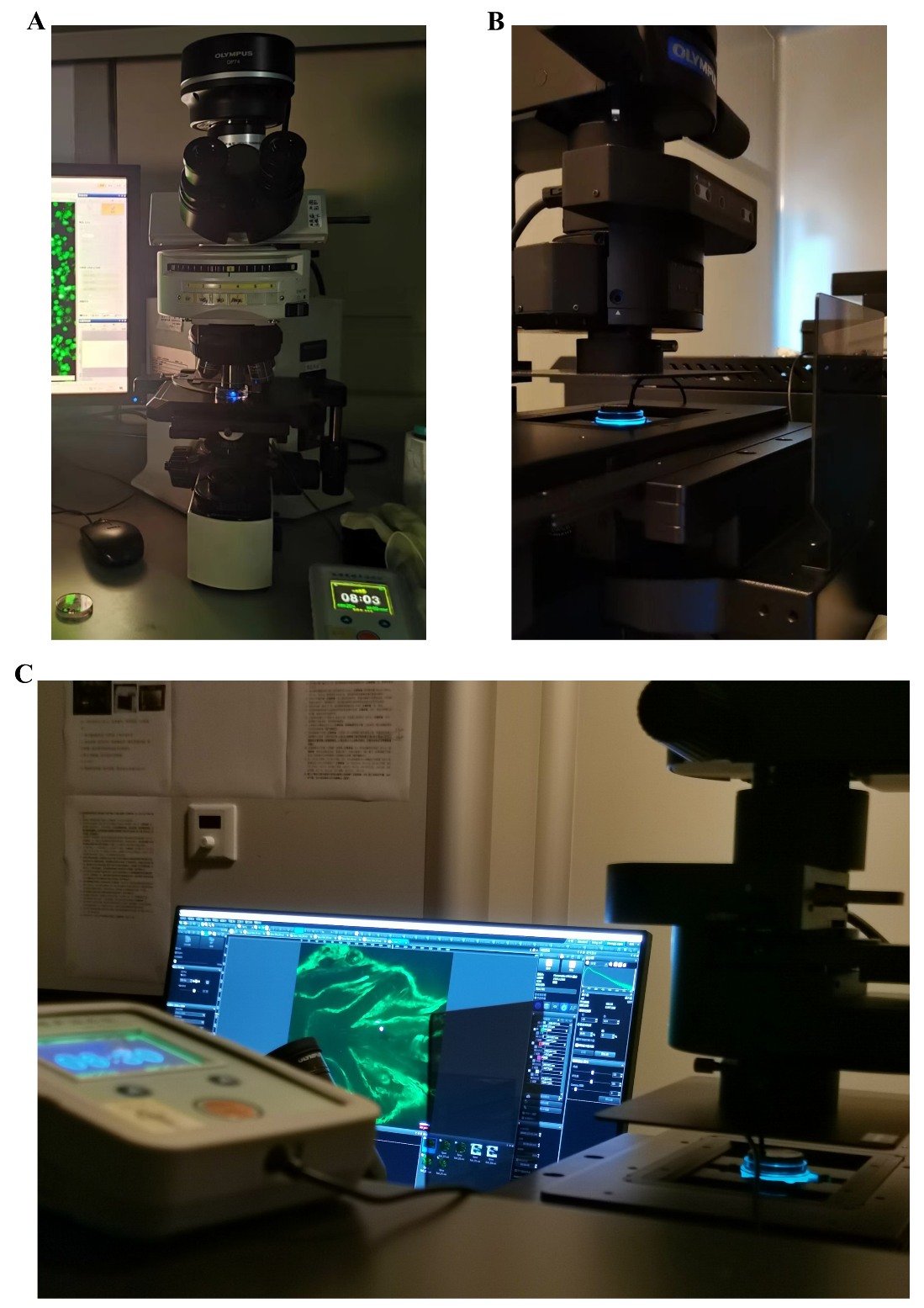


**Figure S1. Observing the calcium signaling in real time under LIPUS treatment.** (**A**) A device for real-time observation in vitro. (**B**) and (**C**) A device for real-time observation in vivo.


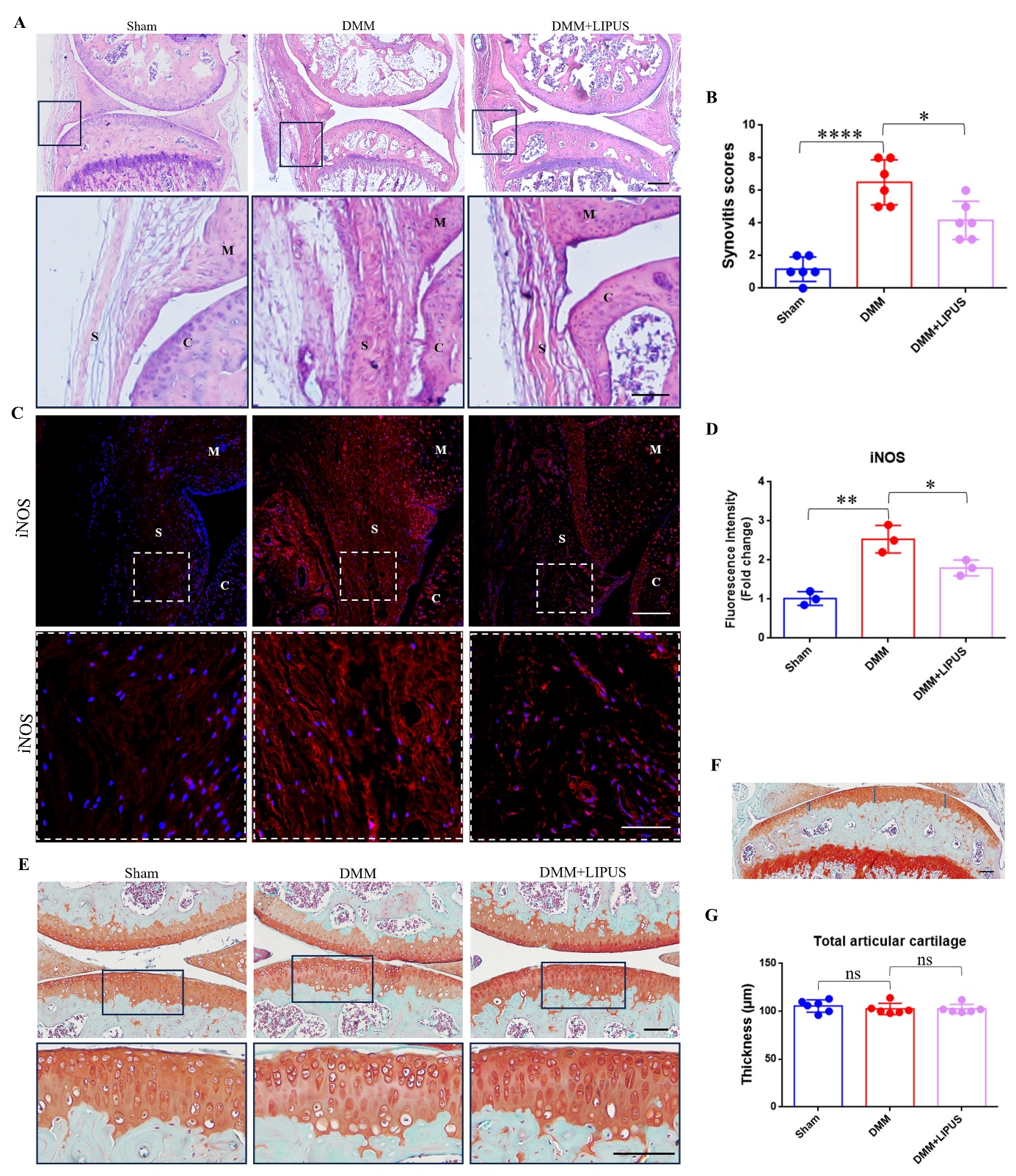


**Figure S2. The synovial inflammation and cartilage thickness at two weeks post-DMM surgery.** (**A**) HE staining of the knee joint at 2 weeks post DMM. Scale bar, 100μm. (**B**) The total synovitis scores of each group was evaluated (n=6 mice). (**C**) Immunofluorescence was used to detect the expression of iNOS in synovium of each group. Scale bars: 100 μm (upper) and 50 μm (lower). (**D**) Quantification the relative fluorescent intensity of iNOS in synovium. (n=3 mice). (**E**) Safranin O–Fast Green staining of the knee joint at 2 weeks post DMM. Scale bar, 100μm. (**F**) Safranin O–Fast Green–stained section of articular cartilage indicating the cartilage regions. Blue lines indicate the approximate locations (quartiles 1/2/3 of the surface of the entire articular cartilage) at which measurements of articular cartilage thickness were taken, Scale bar, 100μm. (**G**) Quantification the mean total of articular cartilage thickness (n=6 mice).


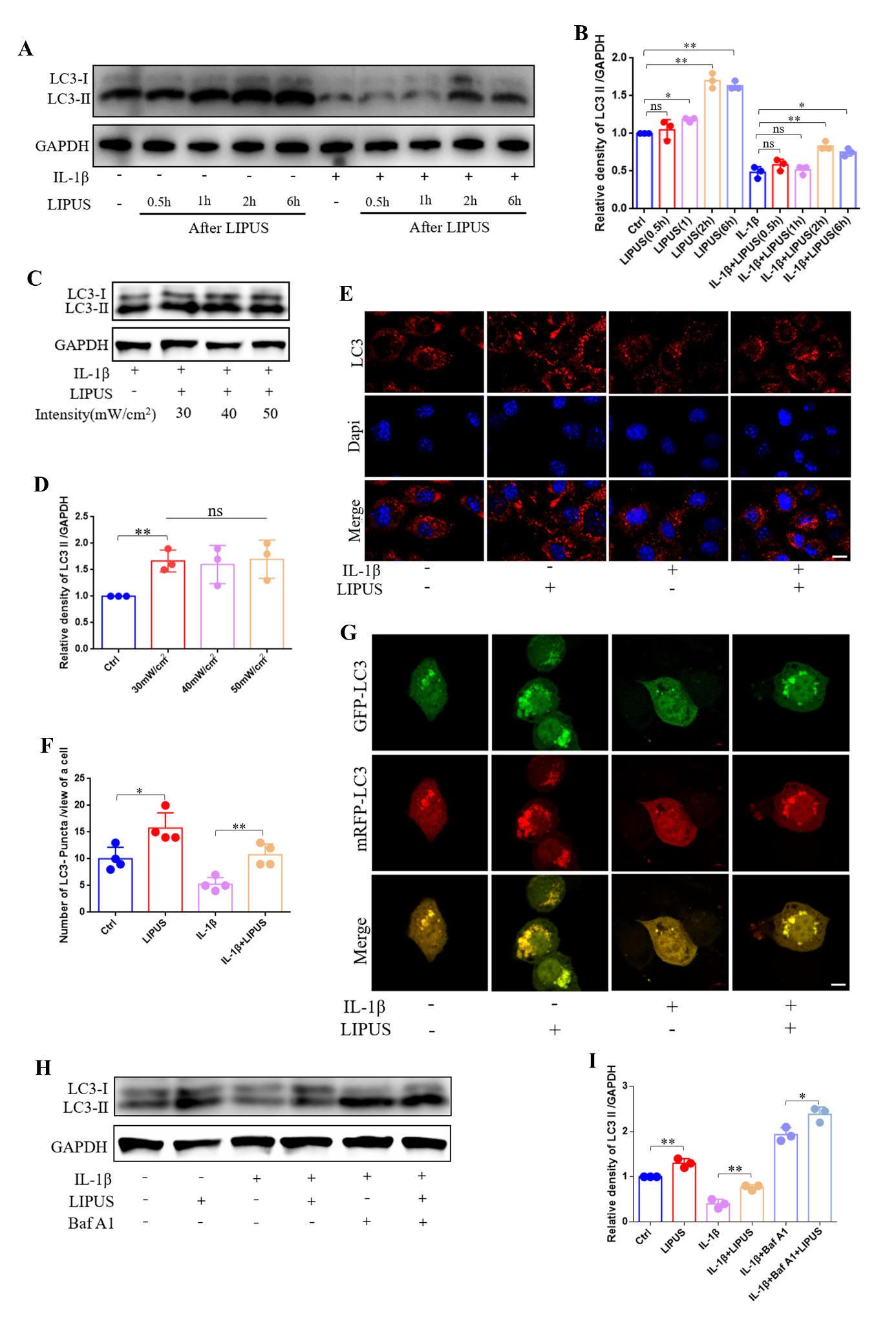


**Figure S3. LIPUS up-regulates chondrocyte autophagy.** WB (**A**) and densitometry analysis of LC3 (**B**) protein expression of chondrocyte at different time point after LIPUS treatment (n=3). WB (**C**) and densitometry analysis of LC3 (**D**) protein expression of chondrocyte following different intensity of LIPUS treatment (n=3). (**E**) Immunofluorescence was used to detect the LC3-puncta of chondrocytes following LIPUS. Scale bar, 10μm. (**F**) Quantification of LC3-puncta in chondrocytes (n=4). (**G**) Confocal images of chondrocytes stably expressing mRFP-GFP-LC3 following LIPUS. Scale bar: 5 μm. WB (**H**) and densitometry analysis of LC3 (**I**) protein expression of chondrocyte co-incubated with Baf A1 following LIPUS treatment (n=3). Data are presented as means ± SD. Statistical analysis was performed using Student’s t test. **(P < 0.01), *(P< 0.05), ns (0.05 < P).


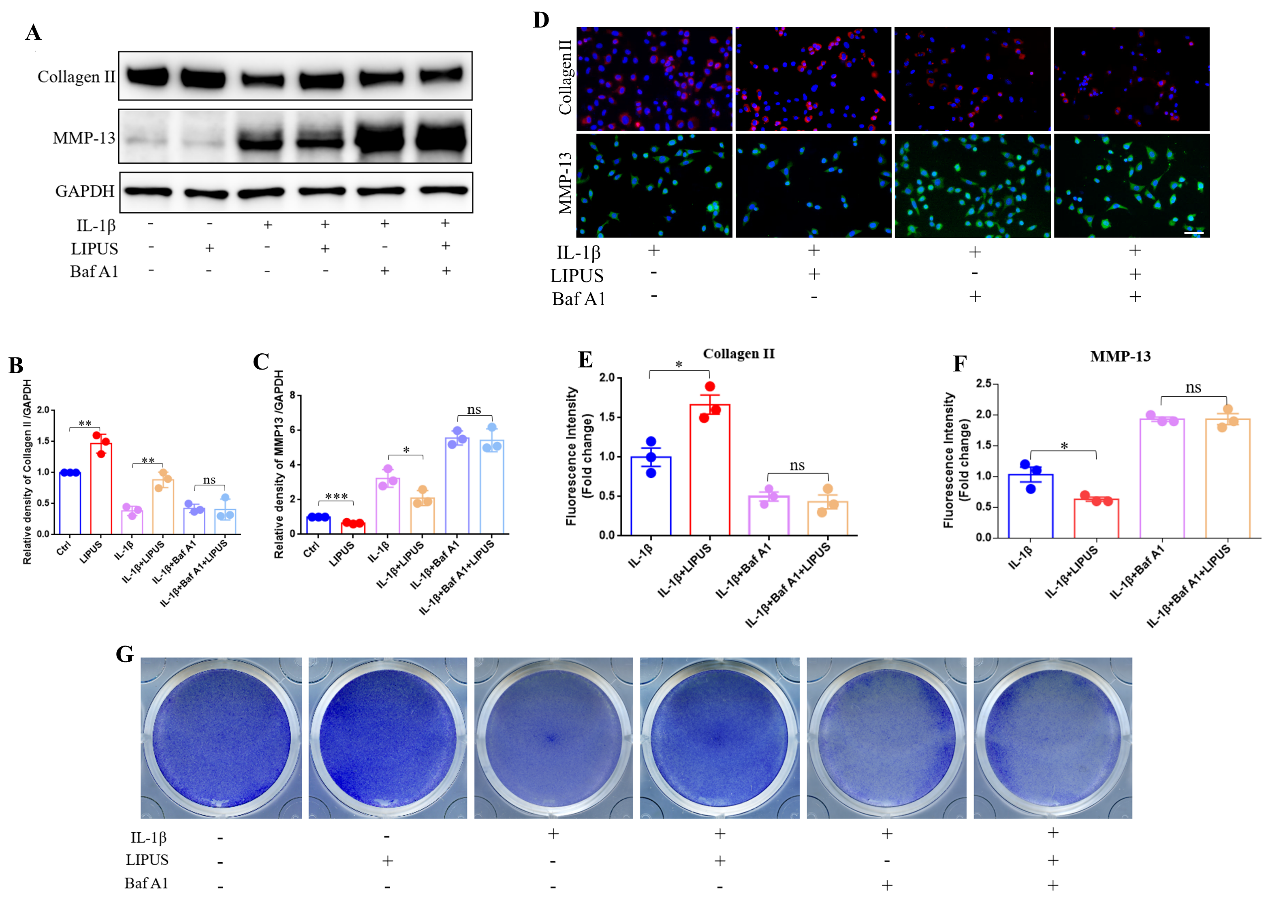


**Figure S4. LIPUS promotes extracellular matrix anabolic via chondrocyte autophagy.** WB (**A**) and densitometry analysis of Collagen II (**B**) and MMP-13 (**C**) protein expression of chondrocyte co-incubated with Baf A1 following LIPUS treatment (n=3). (**D**) Immunofluorescence was used to detect the expression of Collagen II and MMP-13 after the chondrocytes was co-incubated with Baf A1. Scale bar, 20μm. Quantification the relative fluorescent intensity of Collagen II (**E**) and MMP-13 (**F**) in chondrocytes (n=3). (**G**) Toluidine blue staining was used to detect the anabolic of extracellular matrix of chondrocyte co-incubated with Baf A1 following LIPUS treatment. Data are presented as means ± SD. Statistical analysis was performed using Student’s t test. ***(P < 0.001), **(P < 0.01), *(P<0.05), ns (0.05 < P).


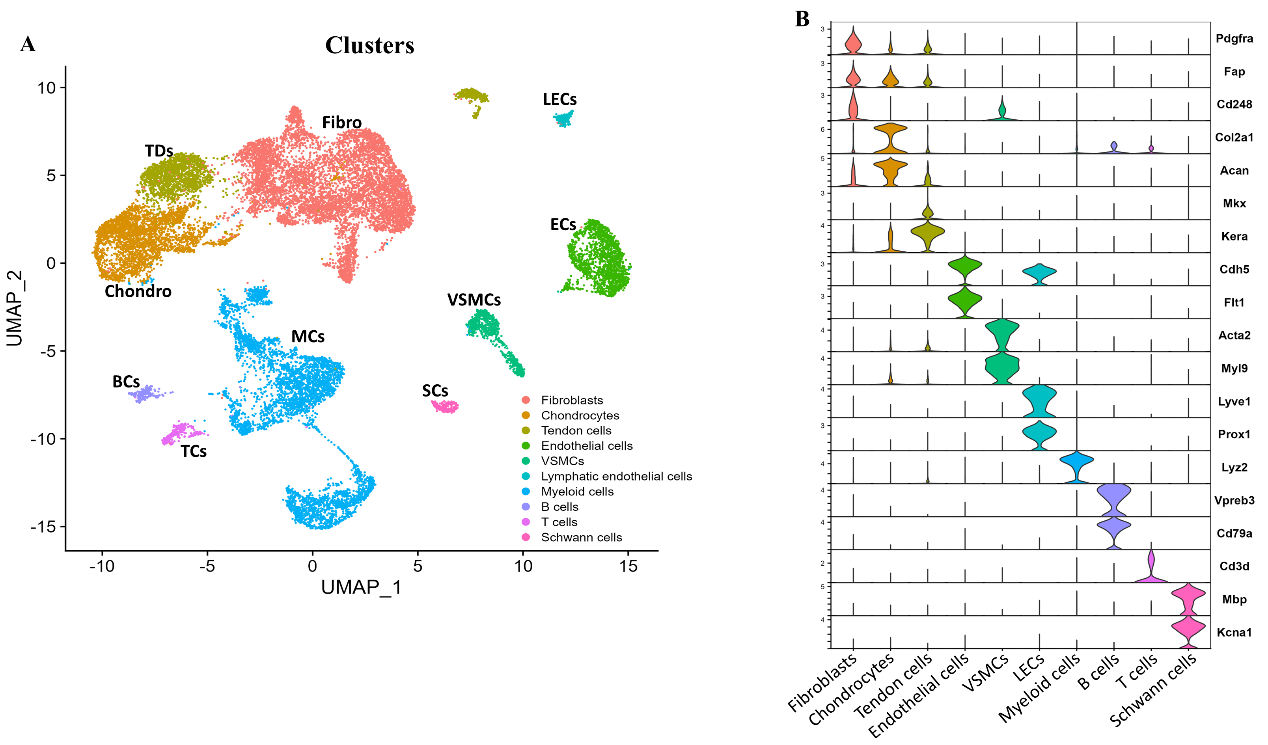


**Figure S5. Transcriptional atlas of joint synovium and cartilage.** (**A**) UMAP plot showing cell types integrated across conditions using canonical correlation analysis. Clusters have designated names and colours corresponding to their annotation (right). (**B**) Violin plots of corresponding markers to cell types.


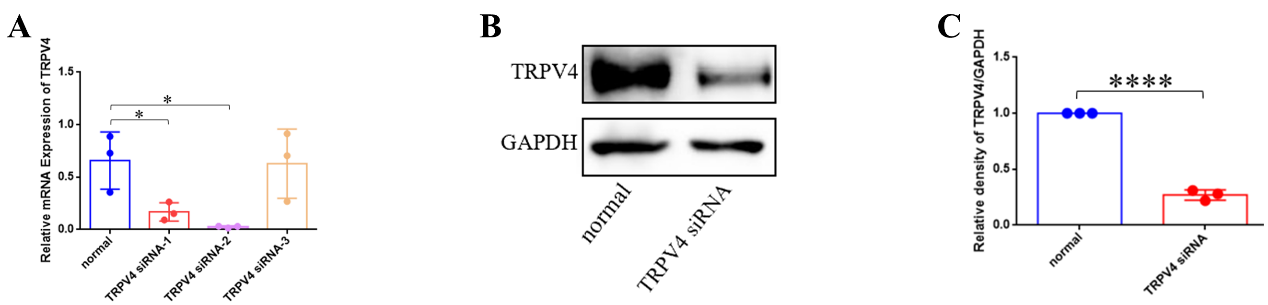


**Figure S6. TRPV4 knockdown in chondrocyte.** (**A**) Relative mRNA expression of TRPV4 was detected by qPCR (n=3). WB (**B**) and densitometry analysis of TRPV4 (**C**) protein expression of chondrocyte after transfection of siTRPV4 (n=3). Data are presented as means ± SD. Statistical analysis was performed using Student’s t test. ****(P < 0.0001), *(P< 0.05).


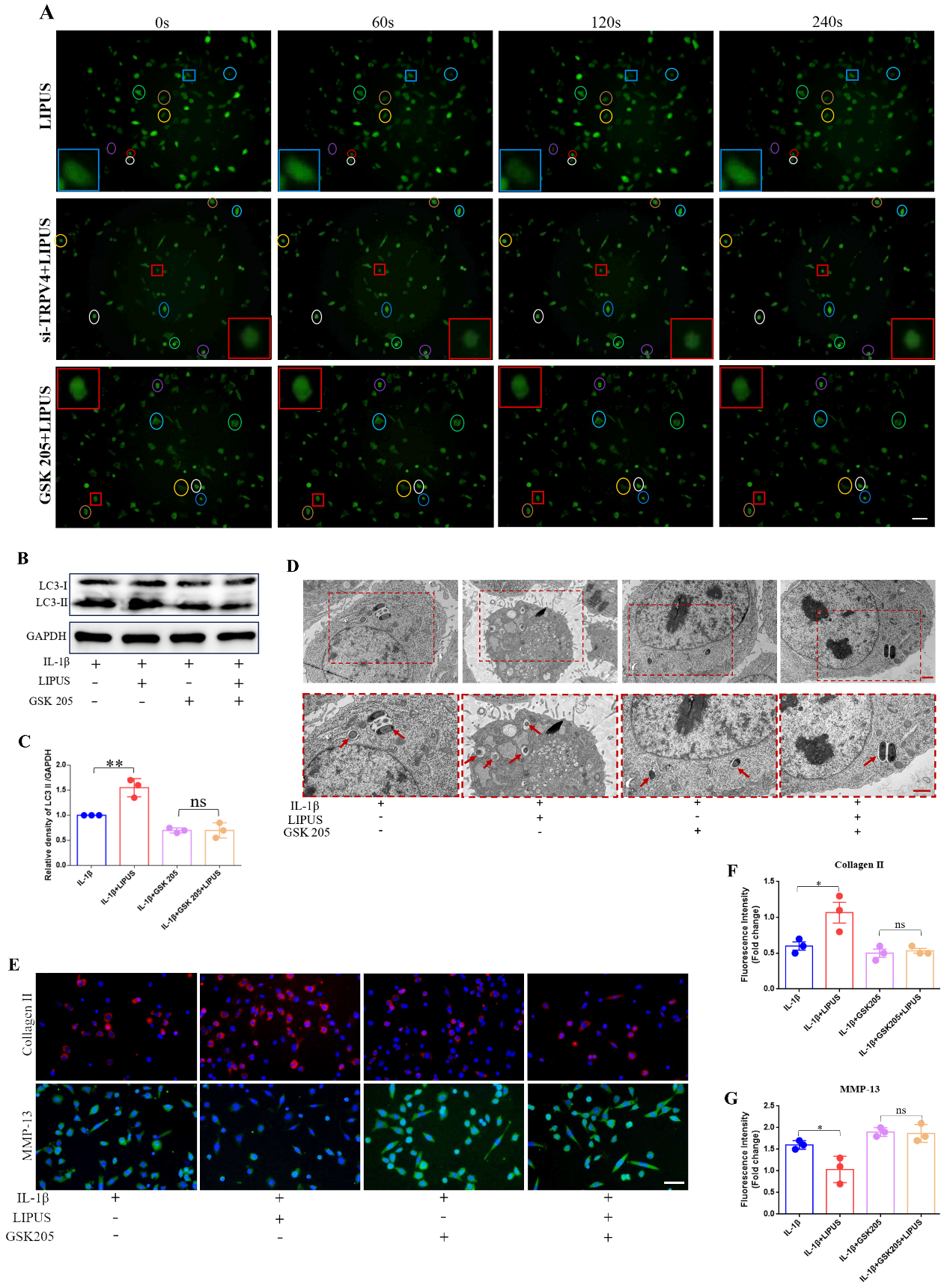


**Figure S7. TRPV4 mediated Ca^2+^ signaling participates the regulation of LIPUS to inflammatory chondrocyte.** (**A**) The real-time fluorescence intensity images of chondrocyte at different time point in the same field of view (Circles of the same color represent the same cell, and square represent the typical cell in this field of view). Scale bar, 10μm. WB (**B**) and densitometry analysis of LC3 (**C**) protein expression of chondrocyte co-incubated with GSK205 following LIPUS treatment (n=3). (**D**) TEM was used for detection the autophagosomes in chondrocytes co-incubated with GSK205 following LIPUS treatment. Red arrowhead indicates an autophagosome. Scale bar, 1μm. (**E**) Immunofluorescence was used to detect the expression of Collagen II and MMP-13 after the chondrocytes was co-incubated with GSK205. Scale bar, 20μm. Quantification the relative fluorescent intensity of Collagen II (**F**) and MMP-13 (**G**) in chondrocytes (n=3). Data are presented as means ± SD. Statistical analysis was performed using Student’s t test. **(P < 0.01), *(P< 0.05), ns (0.05 < P).


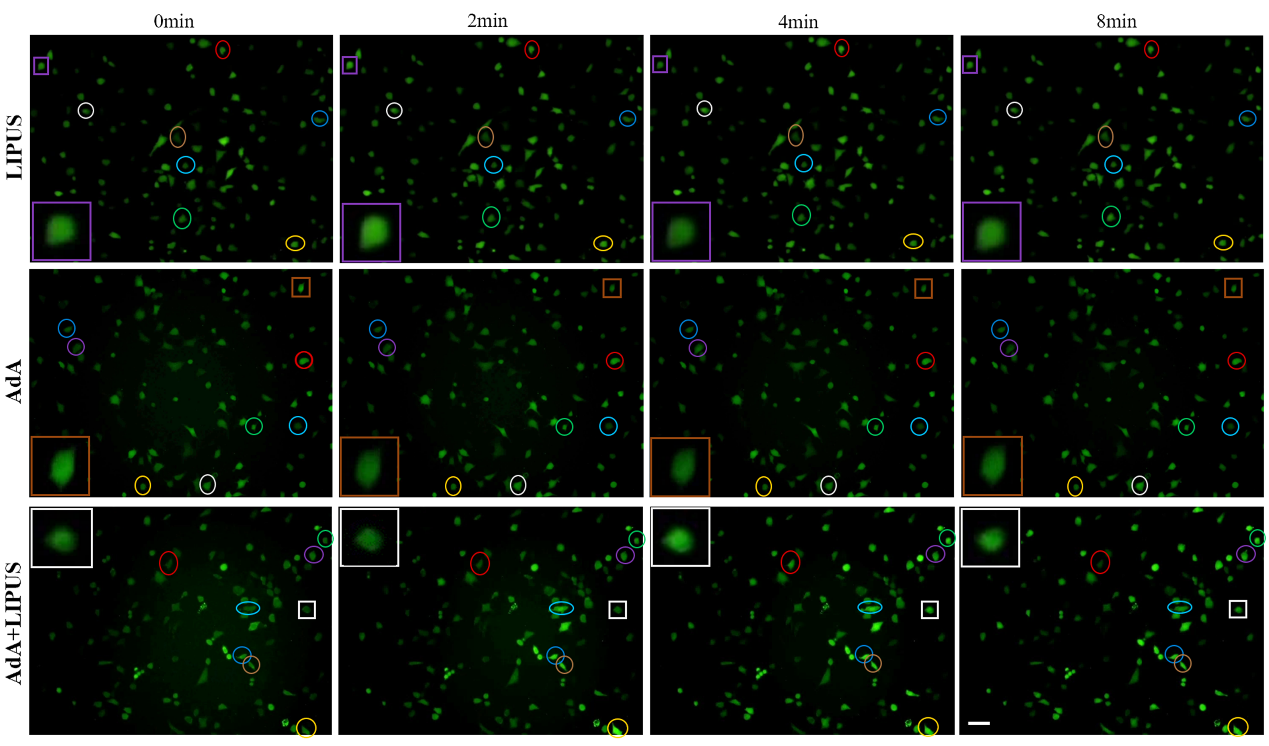


**Figure S8. The intracellular calcium agonist AdA has a synergistic effect with LIPUS in activating Ca^2+^ signaling of inflammatory chondrocyte.** The real-time fluorescence intensity images of chondrocyte in LIPUS alone, AdA alone and in the combination of LIPUS and AdA (Circles of the same color represent the same cell, and square represent the typical cell in this field of view). Scale bar, 10μm.
